# Emodin reduces Breast Cancer Lung Metastasis by suppressing Macrophage-induced Breast Cancer Cell Epithelial-mesenchymal transition and Cancer Stem Cell formation

**DOI:** 10.7150/thno.45395

**Published:** 2020-07-09

**Authors:** Qing Liu, Johnie Hodge, Junfeng Wang, Yuzhen Wang, Lianming Wang, Udai P. Singh, Yong Li, Yongzhong Yao, Dawei Wang, Walden Ai, Prakash Nagarkatti, Hexin Chen, Peisheng Xu, E. Angela Murphy, Daping Fan

**Affiliations:** 1 Department of Cell Biology and Anatomy, University of South Carolina School of Medicine, Columbia, SC 29209.; 2 Department of Statistics, University of South Carolina, Columbia, SC 29208.; 3 Department of Pathology, Microbiology and Immunology, University of South Carolina School of Medicine, Columbia, SC 29209.; 4 Department of General Surgery, Nanjing Drum Tower Hospital, the Affiliated Hospital of Nanjing University Medical School, Nanjing, China, 210008.; 5 Guangdong Provincial Hospital of Chinese Medicine, the 2nd Clinical School of Medicine, Guangzhou University of Chinese Medicine, Guangzhou, 510120, China.; 6 Department of Biology and Environmental Health Science, Benedict College, Columbia, SC 29204.; 7 Department of Biological Sciences, University of South Carolina, Columbia, SC 29208.; 8 Department of Drug Discovery and Biomedical Sciences, University of South Carolina, College of Pharmacy, Columbia, SC 29208.

**Keywords:** Breast cancer, Emodin, Macrophage, Epithelial-mesenchymal transition, Cancer stem cell

## Abstract

Our previous studies demonstrated that the natural compound emodin blocks the tumor-promoting feedforward interactions between cancer cells and macrophages, and thus ameliorates the immunosuppressive state of the tumor microenvironment. Since tumor-associated macrophages (TAMs) also affect epithelial mesenchymal-transition (EMT) and cancer stem cell (CSC) formation, here we aimed to test if emodin as a neoadjuvant therapy halts breast cancer metastasis by attenuating TAM-induced EMT and CSC formation of breast cancer cells.

**Methods:** Bioinformatical analysis was performed to examine the correlation between macrophage abundance and EMT/CSC markers in human breast tumors. Cell culture and co-culture studies were performed to test if emodin suppresses TGF-β1 or macrophage-induced EMT and CSC formation of breast cancer cells, and if it inhibits breast cancer cell migration and invasion. Using mouse models, we tested if short-term administration of emodin before surgical removal of breast tumors halts breast cancer post-surgery metastatic recurrence in the lungs. The effects of emodin on TGF-β1 signaling pathways in breast cancer cells were examined by western blots and immunofluorescent imaging.

**Results:** Macrophage abundance positively correlates with EMT and CSC markers in human breast tumors. Emodin suppressed TGF-β1 production in breast cancer cells and macrophages and attenuated TGF-β1 or macrophage-induced EMT and CSC formation of breast cancer cells. Short-term administration of emodin before surgery halted breast cancer post-surgery metastatic recurrence in the lungs by reducing tumor-promoting macrophages and suppressing EMT and CSC formation in the primary tumors. Mechanistic studies revealed that emodin inhibited both canonical and noncanonical TGF-β1 signaling pathways in breast cancer cells and suppressed transcription factors key to EMT and CSC.

**Conclusion:** Natural compound emodin suppresses EMT and CSC formation of breast cancer cells by blocking TGF-β1-mediated crosstalk between TAMs and breast cancer cells. Our study provides evidence suggesting that emodin harbors the potential for clinical development as a new effective and safe agent to halt metastatic recurrence of breast cancer.

## Introduction

Most breast cancer deaths are resulted from metastatic recurrence after initial success of surgery and/or other therapies 1, 2. Neoadjuvant and adjuvant therapies have been increasingly used, particularly for triple-negative breast cancer (TNBC) and HER2-positive breast cancer 3, in order to prevent local or distant tumor recurrence after surgery. However, the metastatic rate of breast cancer is still substantial 4, and chemotherapy-associated side effects compromise quality of life. Therefore, more effective and safer neoadjuvant or adjuvant treatments are urgently needed. Natural compounds are an eminent source of drug development; many blockbuster drugs, such as paclitaxel and artemisinin, are plant extracts 5, 6. We aim to develop a pleiotropic natural compound, emodin (1,3,8-trihydroxy-6-methylanthraquinone), as an effective and safe agent to halt breast cancer post-surgery metastatic recurrence.

Epithelial-mesenchymal transition (EMT) contributes to cancer progression, and particularly, metastasis 7. Reprogramming of gene expression during EMT is initiated and controlled by various signaling pathways in response to extracellular cues, among which the transforming growth factor-β (TGF-β) signaling plays a predominant role 8. The self-renewing cancer stem cells (CSCs) and progenitor cells constitute a minor portion of neoplastic cells within the tumor and are collectively defined as tumor-initiating cells (TIC) 9. The TIC population is the key source of metastatic lesions in breast cancer 10, 11. The relationship between EMT and CSC is well documented; EMT confers cancer cell mesenchymal traits and an ability to enter the CSC state 12-14. Furthermore, EMT promotes tumor cell invasion and dissemination; it also enables CSCs to clonally expand in distant organs 8, leading to cancer metastasis.

Emodin is an anthraquinone derivative isolated from many Chinese herbs including *Rheum palmatum L.* and *Polygonum cuspidatum.* Our previous studies have shown that emodin blocks the tumor-promoting feedforward interactions between cancer cells and macrophages, reduces recruitment of macrophages to the tumor and their subsequent M2-like polarization, and thus ameliorates the immunosuppressive state of the tumor microenvironment (TME) 15-17. When emodin was administered to mice soon after the tumor cells were inoculated, it inhibited breast tumor growth 16; while when emodin treatment began after tumors that were well established; it had no effects on the growth of the primary tumor but significantly reduced lung metastasis 17. Because tumor-associated macrophages (TAMs) also promote EMT of cancer cells and the generation of CSCs, contributing to cancer invasion and metastasis 18-20, we hypothesize that emodin inhibits breast cancer cell EMT and reduces CSC through acting on both macrophages and cancer cells, and thus halts breast cancer post-surgery metastatic recurrence if it is administered as a neoadjuvant therapy.

## Methods

### Mice

Mice including C57BL/6, BALB/c, and NOD-SCID mice were purchased from Jackson Laboratories. MMTV-PyMT mice generated on an FVB background 21 were crossed to the C57BL/6 background in Dr. Zena Werb's laboratory at UCSF and further in our lab for over 10 generations. All mice were housed in the University of South Carolina Department of Laboratory Animal Research. Animal care procedures and experimental methods were approved by the Institutional Animal Care and Use Committee of the University of South Carolina according to National Institutes of Health guidelines.

### Cell culture

The breast cancer cell lines EO771, 4T1, MCF7, and MDA-MB-231 were obtained from the American Type Culture Collection. The cell line authentication was described in our recent study 22. Cells were cultured in high glucose Dulbecco's modified Eagle medium (DMEM, Invitrogen) with 10% FBS (Invitrogen) and penicillin/streptomycin at 37°C in a humidified 5% CO_2_ incubator.

### Primary cell isolation

To obtain primary MMTV-PyMT cells, mouse mammary tumors were cut into small fragments (<3 mm) and digested in dissociation solution (DMEM supplemented with 10% FBS, Collagenase type IV (5320 U), DNase I (319 U) and hyaluronidase (500 U)) for 60 min in a 37°C water bath with shaker. After digestion and filtering, erythrocytes were lysed with red blood cell lysing buffer (Sigma). Cell suspensions were passed through 70-μm cell strainers; cells were then washed and cultured in complete medium for further experimentation.

### Collection of cell conditioned medium

To obtain tumor cell conditioned medium (TCCM) or peritoneal macrophage conditioned medium (PMCM), the tumor cells (4T1 or EO771) were cultured to 90% confluence in complete medium, and mouse peritoneal macrophages were isolated from mice as described previously 22 and cultured in the indicated medium overnight, and then the medium was replaced with serum-free DMEM. After 24 h, the medium was collected and filtered through a 0.22 μm filter.

### Coculture of cancer cells with macrophages

The indirect contact coculture was performed in 24-well plates with 8 μm polyethylene terephthalate membrane filters (Corning) separating the lower and upper chambers. After the pretreatment with corresponding TCCM with or without emodin at indicated concentrations, macrophages from syngeneic mice were seeded in the upper chambers, while EO771 cells or 4T1 cells were seeded in the lower chamber; 48 h later, cancer cells in the lower chamber were collected for analysis.

### Wound-healing migration assay and invasion assay

The cell migration margins before and after stimulation were marked and the distances were calculated by image processing software Image-Pro Plus 6.0. The invasion of breast cancer cells was examined using 24-well Matrigel-coated invasion chambers with 8 μm pore size inserts (Corning). Cell migration was allowed for 24 h. Non-invading cells on the upper surface of the inserts were removed from the chambers and cells that reached the lower surface were fixed and stained by DAPI. The invaded cells were counted and quantified with the image processing software Image-Pro Plus 6.0.

### Aldefluor assay

The Aldefluor assays were conducted using the Aldefluor assay kit following the manufacturer's instructions (STEMCELL Tech). Briefly, Aldefluor reagents were added to the cell suspension after indicated treatments. Cell plates were incubated at 37°C for 30 min. After washing, flow cytometry analysis was conducted to quantify ALDH^br^ cells. Cells treated by an ALDH inhibitor, diethylamino benzaldehyde (DEAB), were used as a negative control.

### Tumor mammosphere formation assay

A primary mammosphere formation assays were performed in ultra-low attachment 96-well plates (Corning Costar). Dissociated breast cancer cells (EO771, 4T1, MCF7, or MDA-MB-231) at indicated numbers (3000, 1000, 300, 100 and 30 cells) were cultured in serum-free DMEM with 20 ng/ml EGF, 20 ng/ml bFGF and 1 × B27 supplement. On Day 7 of culture, the numbers of primary mammospheres with a diameter larger than 50 μm were counted, and cells were serially passaged for secondary mammosphere formation for another 7 days.

### *In vivo* limiting dilution analysis

Cancer cells pretreated with emodin or DMSO for 48 h were injected into the 4^th^ pair of mammary fat pads in mice. Tumor cells in each group were implanted with various cell numbers in 20 μl PBS. Approximately four weeks after cell implantation, the tumor numbers were counted, and the tumor-initiating cell frequencies were analyzed using the ELDA software (WEHI).

### Tumor cell inoculation

For orthotopic inoculation of breast cancer cells, 2 × 10^5^ cells in 20 μl PBS were injected into each of the 4^th^ pair of mammary fat pads of mice. For the intravenous injection of breast cancer cells, 1 × 10^6^ cells in 100 μl PBS were injected into the tail vein. To monitor the primary tumor growth, tumors were measured using a caliper at indicated time points, and the tumor volume was calculated using formula: length × width^2^/2.

### Quantitative real-time PCR (qPCR)

Total RNA was extracted using the TRIzol Reagent (Invitrogen) and reverse transcribed using iScript cDNA Synthesis Kit (Bio-Rad, Life Science). qPCR was conducted on a CFX96 system (Bio-Rad) using iQ SYBR Green Supermix (Bio-Rad). All primers used for qPCR analysis were synthesized by Integrated DNA Technologies and the primer sequences are listed in the **Supplemental Table S1**. The relative amount of target mRNA was determined using the comparative threshold (Ct) method by normalizing target mRNA Ct values to those of internal control 18S rRNA. PCR thermal cycling conditions were 3 min at 95°C, and 45 cycles of 15 s at 95°C and 58 s at 60°C.

### Western blot

Cells were lysed in RIPA buffer (Pierce) supplemented with protease inhibitor cocktail and phosphatase inhibitor cocktail (Sigma). The total cellular extract was separated in 10% SDS-PAGE precast gels (Bio-Rad) and transferred onto nitrocellulose membranes (Millipore). Membranes were first probed with indicated primary antibodies, followed by the corresponding secondary antibodies which were conjugated with horseradish peroxidase (Millipore). Protein detection was conducted using ECL substrate (Pierce) and the signal intensities were quantified by Image-Pro Plus 6.0.

### Flow cytometry

A single cell suspension was made from cultured cells or mouse tissue after enzyme digestion. For the staining of cell surface markers, cells were blocked in Fc blocker antibody and then stained with indicated antibodies conjugated with a fluorescent dye in staining buffer (PBS containing 2% FBS) for 30 min on ice and in the dark. Samples were washed twice with staining buffer, and then analyzed by a FACS Aria flow cytometer (BD).

### Immunofluorescence staining

For cell slides, cells were fixed in 4% paraformaldehyde and washed with PBS. After blocking, anti-mouse or anti-human primary antibodies were applied to the cell slides overnight at 4°C. Then the slides were washed and incubated with appropriate secondary antibodies in 1% BSA for 1 h at room temperature in the dark, followed by washing, mounting and sealing with a coverslip before observation under a fluorescence microscope (Nikon). A series of images from each sample were taken at the same photography setting, and the mean fluorescence intensity (MFI) was measured by Image-Pro Plus 6.0. For breast tumor tissues from patients, de-identified formalin-fixed paraffin embedded tissues were collected from mastectomy surgery with ethical approval by Nanjing Drum Tower Hospital in 2015. Sections were cut (4-μm thick), transferred to a warm water bath, and placed on a glass slide. Immunofluorescence staining was performed using anti-human antibodies: p-Smad2 (1:300, Cell Signaling) and CD68 (1:300, Abcam).

### Database mining

The cancer genome atlas data repository was used as the primary source of samples for the analysis. Dimensionality reduction was performed with principal component analysis (PCA). Three representative samples in each of the CD68^lo^ and CD68^hi^ groups were used for a heatmap plot of the differentially expressed genes. Correlations between the expression levels of CD68 and the indicated genes were calculated by the spearman correlation coefficient and statistical significance was shown as -log (P value). For the prognostic analysis of TGFβR1 and Smad2, the breast cancer patient survival data of TCGA was obtained from the Human Protein Atlas database (https://www.proteinatlas.org). Based on the median value (FPKM) of each gene, patients were classified into two groups and association between survival rate and gene expression was examined. The survival curve was estimated using Kaplan-Meier analysis, and the P-values were calculated with the log-rank (Mantel-Cox) test.

### Statistical analysis

Data were presented as mean ± SEM. Statistical significance was calculated using the Student* t* test (two-tailed, for two-group comparison) or *one-way ANOVA* followed by post *Dunnett's* test (for multi-group comparison) using the GraphPad Prism statistical program. Survival was analyzed using the *Log-rank (Mantel-Cox)* test. *P* < 0.05 was considered as statistically significant.

## Results

### Macrophage abundance correlates with EMT and CSC markers in human breast tumors

**Figure 1:**
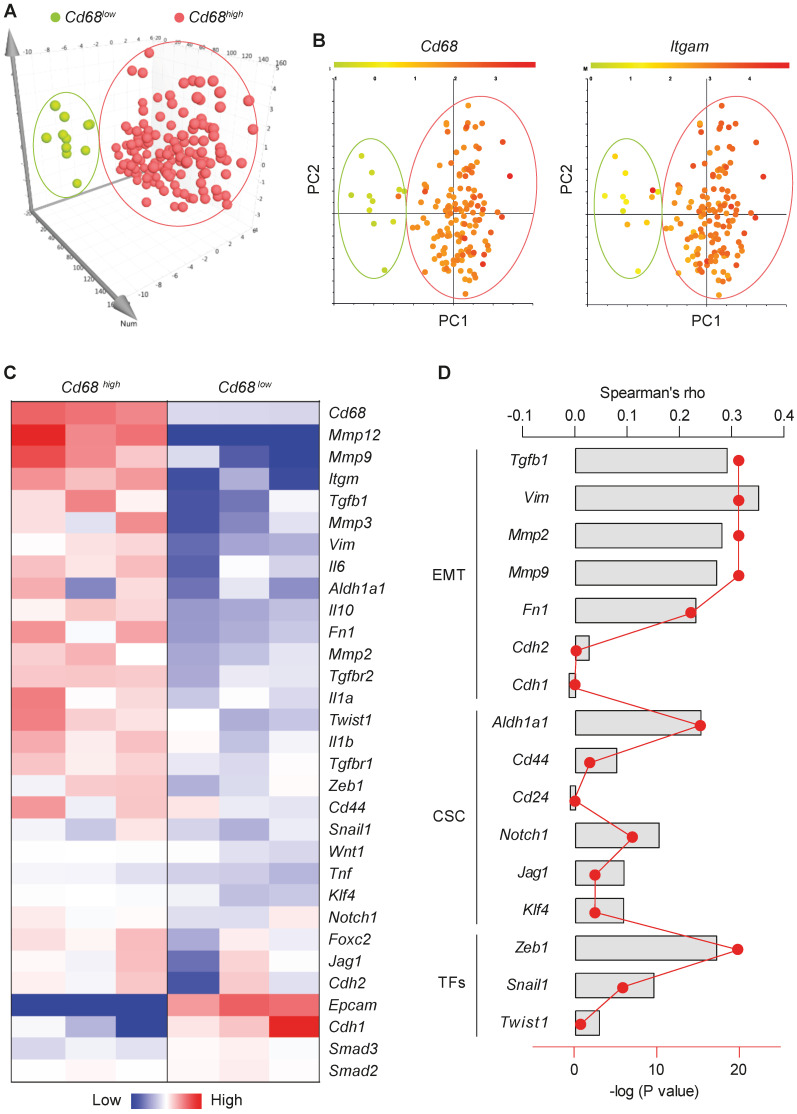
** Macrophage abundance correlates with EMT and CSC markers in human breast tumors. A.** Principal component analysis (PCA) of the human microarray data from the TCGA database. Each dot represents one sample. N = 147 sorted from 529 samples, based on the CD68 expression;** B.** CD68 and ITGAM expression patterns of the PCA-categorized groups from A. N = 147;** C.** Heatmap of the differentially expressed genes after clustering. Three representative samples from each of CD68^lo^ and CD68^hi^ groups were used for plotting; **D.** Spearman correlation analysis of CD68 expression and the expression of indicated genes, including TGF-β1, EMT markers, CSC markers and associated transcription factors. Columns were used to indicate Spearman correlation coefficient (rho), while the dot-line plot indicates the -log (P value).

Macrophages, the most abundant leukocytes in mammary tumors, play critical roles in cancer progression 23. TAMs exhibit a high plasticity and remodel the TME in response to various signals including those from tumor cells 24. To examine the relationship between macrophage abundance and other properties in breast tumors, data from 1,105 samples obtained from 1,098 breast cancer patients in the cancer genome atlas were examined. Among them, microarray data are available for 529 samples. Using the expression level of the pan-macrophage marker CD68 as an indicator of macrophage abundance, we sorted the CD68^lo^ and CD68^hi^ samples (cutoff, fold change > 1.5; n = 147). Dimensionality reduction with PCA clearly categorized CD68^lo^ and CD68^hi^ samples as two separate groups (**Figure 1A**). The expression pattern of another common marker of macrophages, ITGAM (CD11b), in those samples is very similar to that of CD68 (**Figure 1B**). Using three representative samples from each of the CD68^lo^ and CD68^hi^ groups, visualization of heatmap of differentially expressed mRNAs strongly demonstrated that CD68^lo^ and CD68^hi^ groups displayed distinct mRNA expression patterns (**Figure 1C**). We examined the overall expression pattern of these genes in all of the sorted samples according to PCA-categorized CD68^lo^ and CD68^hi^ groups, and found that the CD68^hi^ group was associated with pro-tumorigenic markers, including TGF-β1, EMT markers, stemness markers, and related transcription factors (TFs) (**Figure S1**). Further Spearman correlation analysis confirmed the correlation between CD68 expression and the expression of TGF-β1, EMT and CSC markers, as well as the associated TFs (**Figure 1D**). These results suggest that intratumoral macrophages likely play an essential role in EMT and CSC generation. And indeed, a study showed that liposomal simvastatin could suppress cancer cell EMT via repolarizing TAMs 25. Since our previous studies showed that emodin inhibited breast cancer growth and metastasis by blocking the interactions between cancer cells and TAMs 15-17, these bioinformatics data prompted us to investigate if emodin affects EMT and CSC through acting on TAMs in the TME.

### Emodin inhibits TGF-β1 and macrophage-induced EMT in breast cancer cells

**Figure 2:**
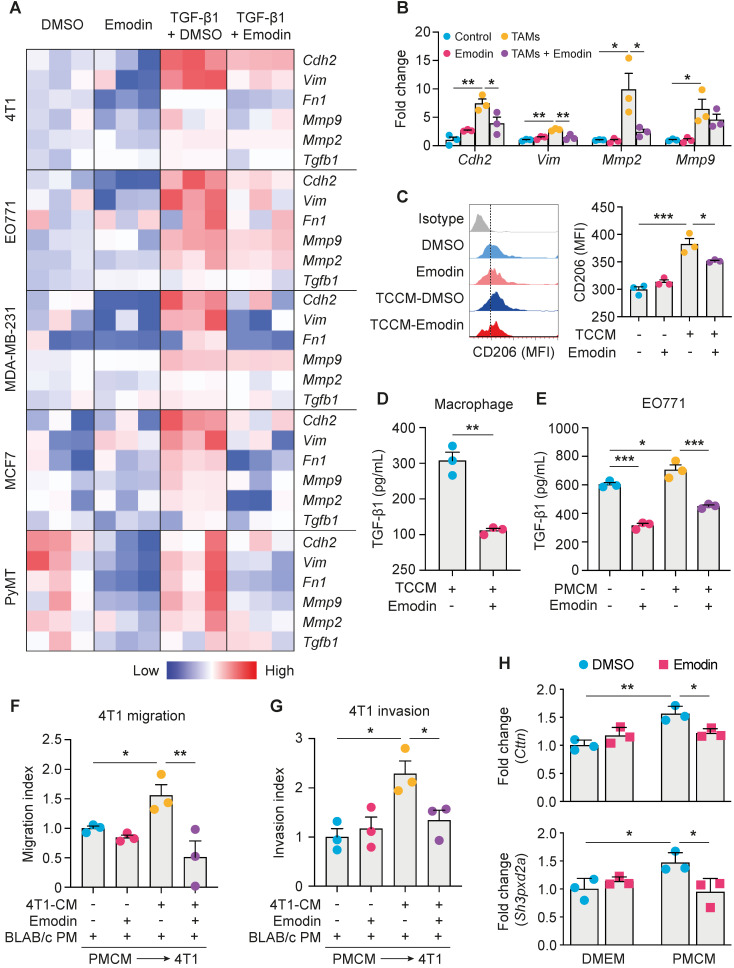
** Emodin inhibits TGF-β1 and macrophage-induced EMT in breast cancer cells. A.** A heatmap plot of emodin-modulated EMT gene expression in breast cancer cells following TGF-β1 stimulation (48 h); data for plotting were obtained by qPCR;** B.** Emodin inhibits EMT gene expression in 4T1 cells cocultured with TAM-like macrophages; *P<0.05; **P<0.01;** C.** Flow cytometry analysis of CD206 expression in TCCM-induced macrophages, with or without emodin treatment; **D-E.** ELISA measurement of TGF-β1 production from macrophages (**D**) or breast cancer cells (**E**) after the indicated stimulation (PMCM: peritoneal macrophage conditioned medium). *P<0.05; **P<0.01; ***P<0.001;** F.** Wound-healing assay of the 4T1 cell migration following PMCM stimulation with or without emodin treatment (16 h). Migration index = (Width before migration - Width after migration)/ Width before migration; all data were normalized to the first group; *P<0.05; **P<0.01; representative images are shown in Figure S5A. **G.** Matrigel invasion assay of 4T1 cells following PMCM treatment with or without emodin. Matrigel concentration: 300 µg/ml; Transwell pore size: 8 µm; Invasion time: 24 h; Invasion index was calculated by the normalization of cell number per field in each group to the DMSO control group. *P<0.05; representative images of the invade cells staining with DAPI are shown in Figure S5B**. H.** Gene expression of the invadopodia formation markers in breast cancer cells. EO771 cells were treated with vehicle or emodin, or conditioned medium from macrophages treated with EO771 conditioned medium with or without emodin (16 h). *P<0.05; **P<0.01.

First, we used the TGF-β1-induced EMT model to assess the effects of emodin on breast cancer cell EMT. It was found that TGF-β1 induced 4T1 cells to acquire a fibroblast-like, mesenchymal cell morphology (**Figure S2A**), accompanied with increased expression of signature genes of EMT (**Figure S2B**). The inhibitory effect of emodin on EMT was tested using various breast cancer cell lines (**Figure S2C-G**). In all tested breast cancer cell lines, upregulation of EMT genes by TGF-β1, including TGF-β1 itself, was diminished by emodin (**Figure 2A**). Loss of the epithelial marker E-cadherin and acquisition of the mesenchymal marker N-cadherin are key features of the EMT process 26. Western blotting showed that TGF-β1 treatment increased N-cadherin and decreased E-cadherin protein levels in various breast cancer cell lines, while emodin reversed these changes (**Figure S3A**). Immunofluorescence staining confirmed that the TGF-β1-induced loss of E-cadherin was reversed by emodin (**Figure S3B-C**).

Cancer cells and TAMs at the invasive front of breast tumors interact to enable cancer cell invasion 27. To determine whether emodin affects macrophage-induced EMT of breast cancer cells, we isolated primary macrophages from mice and pretreated them with tumor cell conditioned medium (TCCM) to generate TAM-like macrophages, and then added them to breast cancer cells (**Figure S4A**). Examination of the expression of EMT markers indicated that emodin significantly attenuated TAM-like macrophage-induced N-cadherin, Vimentin and MMP expression in breast cancer cells (**Figure 2B and S4B**). Based on our previous findings 16, we hypothesized that emodin may suppress TCCM-induced macrophage M2-like polarization and M2-like macrophage-induced EMT of breast cancer cells by blocking the TGF-β1-mediated reciprocal interaction between macrophages and cancer cells. As expected, TCCM-pretreated macrophages exhibited an increased expression of CD206, a marker of M2 macrophages, which was attenuated by emodin (**Figure 2C**). ELISA showed that TGF-β1 production in macrophages after TCCM treatment was significantly decreased by emodin (**Figure 2D**). In addition, while TGF-β1 production in breast cancer cells was elevated after peritoneal macrophage conditioned medium (PMCM) treatment, emodin significantly reduced baseline and PMCM-induced TGF-β1 production in cancer cells (**Figure 2E**). Moreover, we confirmed by qPCR that emodin suppressed both baseline and TGF-β1 induced Arg-1 expression in macrophages (Figure **S4C**); Arg-1 expression has been shown to define immunosuppressive subsets of TAMs 28. Taken together, these data suggest that emodin 1) suppresses cancer cell-induced macrophage M2-like polarization and thus TGF-β1 production, 2) inhibits polarized macrophage-induced TGF-β1 production in breast cancer cells, and 3) blocks EMT of breast cancer cells induced by TGF-β1 from both macrophages and cancer cells.

### Emodin inhibits TGF-β1 and macrophage-induced migration and invasion of breast cancer cells

EMT enables cancer cells to migrate and invade 29, 30; we thus examined if emodin affects the migration and invasion of breast cancer cells. First, a wound-healing migration assay showed that TGF-β1 enhanced 4T1 breast cancer cell migration by 2-fold, and this effect was abolished by emodin (**Figure S5A**). Second, a matrigel invasion assay demonstrated that TGF-β1 increased 4T1 cell invasion by >5-fold and this effect was significantly diminished by emodin (**Figure S5B**). Similar results were obtained using MDA-MB-231 cells (**Figure S5C** and **S5D**). TAMs aggregate to induce migration, invasion and dissociation of cancer cells through the local production of TGF-β1 and MMPs 31, 32. We thus tested if emodin affects the cancer cell migration and invasion stimulated by conditioned medium of cultured peritoneal macrophages (PMCM). PMCM was collected from macrophages treated with 4T1 conditioned medium with or without emodin. 4T1 cells were then stimulated by PMCM for 16 h, and migration and invasion were measured. 4T1 cells treated with PMCM from macrophages that were treated with 4T1 conditioned medium without emodin exhibited more aggressive migration and invasion compared with those 4T1 cells treated with PMCM from macrophages treated with 4T1 conditioned medium with emodin (**Figure 2F** and** 2G; Figure S5E** and** S5F**).

During breast cancer cell migration and invasion, pioneer cancer cells at the edge of the tumor degrade the extracellular matrix by developing specialized actin-rich membrane protrusion structures called invadopodia 33; and macrophages promote breast cancer cell migration through Notch1-initiated invadopodia formation 34. We thus examined the expression of Cortactin and Tks5, markers of invadopodia formation, following DMEM (with or without emodin) or PMCM treatment. As shown in **Figure 2H**, while emodin itself did not affect Cortactin or Tks5 expression in breast cancer cells, PMCM from macrophages treated with EO771 conditioned medium significantly increased the expression of these two genes; moreover, upregulation of these genes was abolished if the macrophages were treated with emodin in addition to EO771 conditioned medium.

### Emodin suppresses the stemness and progenitor properties of breast cancer cells

**Figure 3:**
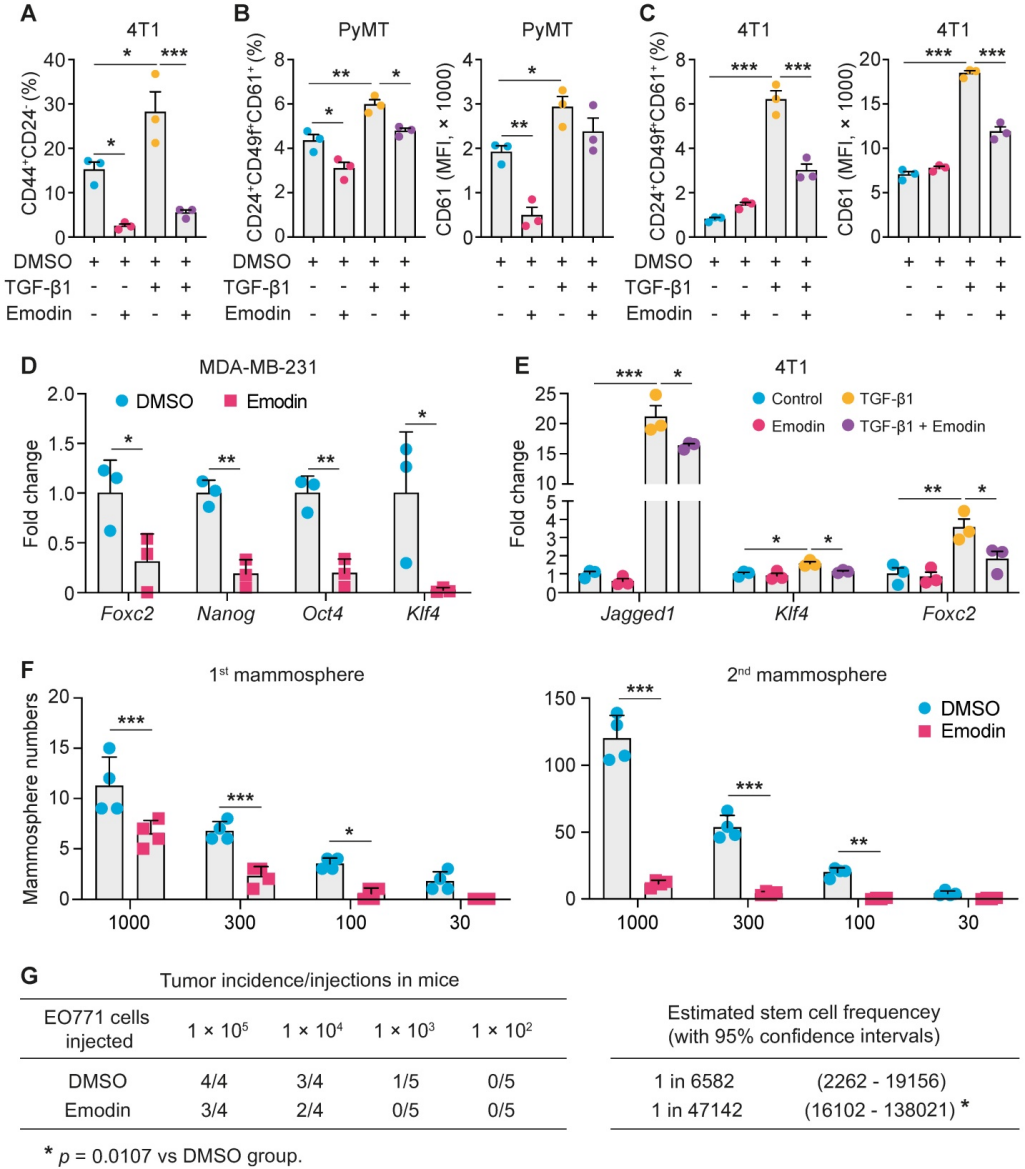
** Emodin suppresses the stemness and progenitor properties of tumor-initiating cells. A.** Flow cytometry analysis of the effect of emodin on cancer stem cell population in 4T1 cells with or without TGF-β1 stimulation (24 h). The cancer stem cell percentages are shown. *P<0.05; ***P<0.001;** B.** Flow cytometry analysis of the effect of emodin on progenitor cell populations in PyMT cells after TGF-β1 stimulation for 48 h; the quantifications of the progenitor cell percentages and the mean fluorescence intensity of CD61 in PyMT cells are shown. The CD61^+^ subpopulation was gated from the CD24^+^CD49f^+^ cell population; *P<0.05; **P<0.01; ***P<0.001;** C.** Flow cytometry analysis of the effect of emodin on progenitor cell populations in 4T1 cells after TGF-β1 stimulation for 48 h; the quantifications of the progenitor cell percentages and the mean fluorescence intensity of CD61 in 4T1 cells are shown. The CD61^+^ subpopulation was gated from CD24^+^CD49f^+^ cell population; ***P<0.001;** D-E.** Emodin inhibited the expression of stemness-related genes in MDA-MB-231 cells (**D**) and 4T1 cells (**E**); data were obtained from qPCR analysis. *P<0.05; **P<0.01; ***P<0.001;** F.** Emodin inhibits tumor mammosphere formation of EO771 cells. The 1^st^ mammospheres were cultured in ultra-low attachment plates for 7 days, and the 2^nd^ mammospheres were cultured for another 7 days; quantification of the number of formed 1^st^ mammospheres and 2^nd^ mammospheres in EO771 culture with or without emodin treatment is shown; the numbers in X-axis indicate the numbers of cells that were added in each well of the plate. **G.** Limiting dilution assays show the inhibitory effect of emodin on breast cancer stem cells in EO771 cells. The data are presented as the number of mice with detectable tumors (0-4) versus total number of mice injected with cancer cells (4 or 5). The estimated stem cell frequencies were calculated for statistical comparison (right).

We hypothesized that emodin also could affect EMT-promoted CSC generation and maintenance. First, we found that emodin reduced baseline levels of CD44^hi/+^/CD24^lo/-^ cells in various breast cancer cell lines (**Figure S6A**); CD44^hi/+^ and CD24^lo/-^ are recognized CSC markers. Measurement of aldehyde dehydrogenase activity (ALDH), another marker of both normal and malignant mammary stem cells 35, confirmed the suppressive effects of emodin on the CSC population in MDA-MB-231 cells (**Figure S6B**). Induction of EMT by TGF-β1 increases breast cancer stem cells 36, 37. We showed that TGF-β1 increased CSCs by 50% in 4T1 cells, while emodin abolished this effect (**Figure 3A and S6C**).

Adult stem cells can generate multipotent progenitors that further develop into specialized cells 38. However, the heterogenic niche in the TME provides WNT and EGF signals that not only help maintain resident stem cells, but also instruct progenitor cells to revert to a stem cell state, contributing to tumor regeneration and therapy resistance 39. We thus further examined if emodin could affect the progenitor population in breast cancer cells using the markers CD24, CD49f and CD61. We found that emodin suppressed the progenitor population in both EO771 and MDA-MB-231 cells (**Figure S6D** and** S6E**). For the primary PyMT tumor cells, emodin suppressed the percentage of progenitor cells and the average CD61 expression levels, with or without TGF-β1 stimulation (**Figure 3B and S6F**). Similarly, TGF-β1 enhanced the percentage of progenitor population and the average CD61 expression level in 4T1 cells, which was halved by emodin (**Figure 3C and S6G**). Additionally, expression levels of transcription factors Oct4, KLF4 and Nanog, and other TIC-associated genes FoxC2 and Jagged1, were determined. We found that the baseline expression of FoxC2, Nanog, Oct4 and KLF4 was downregulated by emodin in MDA-MB-231 cells (**Figure 3D**), and the TGF-β1-induced expression of Jagged1, KLF4 and FoxC2 was decreased by emodin in 4T1 cells (**Figure 3E**).

Breast Cancer TICs can be propagated *in vitro* as nonadherent spheres; these spherical clusters of self-replicating cells formed in suspension cultures are called mammospheres 40, 41. We tested whether emodin affects mammosphere formation. Cultured in the ultra-low attachment plates for 7 days, EO771 cells formed typical mammospheres (primary mammospheres); re-culturing of the cells enzymatically dissociated from the primary mammospheres under the same conditions yielded secondary mammospheres. Emodin significantly reduced both primary and secondary mammosphere formation (**Figure 3F** and**Figure S7A**). Similar results were obtained using 4T1 (**Figure S7B**), MCF7 (**Figure S7C**), and MDA-MB-231 cells (**Figure S7D**).

The effect of emodin on TIC abundance was further investigated *in vivo* using limiting dilution assays 42, 43. An EO771 single-cell suspension was obtained after the cells were pretreated with DMSO or emodin; serial dilutions were prepared and transplanted into the mammary fat pads of C57BL/6 mice. As shown in **Figure 3G**, emodin pretreatment significantly reduced the estimated stem cell frequency. Similar results were obtained using MDA-MB-231 cells in NOD SCID mice (**Figure S7E**). Taken together, both the *in vitro* mammosphere formation assays and the *in vivo* limiting dilution assays demonstrated that emodin reduced breast cancer tumor-initiating cells.

### Emodin modulates immune cell infiltration and inhibits EMT and TICs in breast tumors

**Figure 4:**
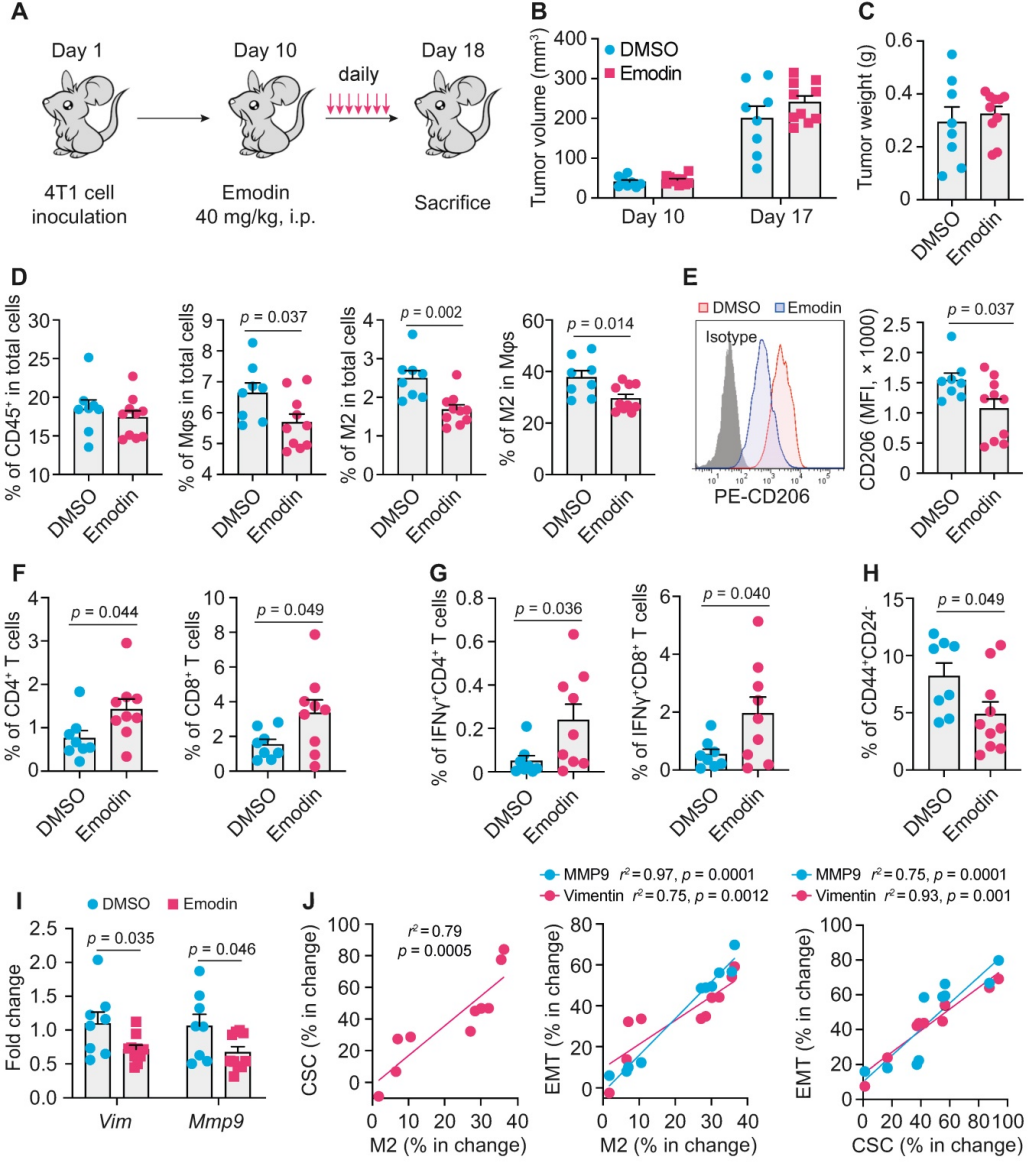
** Emodin enhances anti-tumor immunity and inhibits EMT and CSC in breast tumors. A.** Schematic diagram of the experimental design. After the inoculation of 2 x 10^5^ 4T1 cells per mammary fat pad into the BALB/C mice, emodin (40 mg/kg) or 1% DMSO in PBS was i.p. injected from Day 11 (when tumors became palpable) to Day 17 and then mice were sacrificed on Day 18; **B-C.** Tumor volumes on Day 10 and D17 (**B**) and the tumor weight on Day 18 (**C**) are shown; **D.** Flow cytometry analysis of total macrophages (CD11b^+^F4/80^+^) and CD206^+^ macrophages in primary tumors. Cells were gated from the CD45^+^ cell population (leukocytes) in the total cell suspension. Percentages labeled in gated cells were calculated from total cell population. The percentages of indicated cell populations are shown;** E.** Expression of CD206 (Left) detected by flow cytometry and statistic result (Right); **F-G.** Flow cytometry analysis of total CD4^+^ or CD8^+^ T cells (**F**) and activated CD4^+^ or CD8^+^ T cells (IFNr^+^ cells) (**G**) in primary breast tumors. Cells were gated from CD45^+^ cell population in the total cell suspension. Percentages labeled in gated cells were calculated from total cell population; **H.** Cancer stem cell populations were analyzed by flow cytometry. The stem cell percentages were calculated for comparison (right); **I.** EMT markers in 4T1 tumor tissues were analyzed by qPCR;** J.** Correlation analysis of the changes in M2 macrophage percentage, CSC percentage and EMT markers after emodin treatment compared to DMSO control.

To examine if emodin affects EMT and TICs *in vivo*, we inoculated BALB/c mice with 4T1 cells; after the orthotopic tumors became palpable, mice were treated with emodin daily for 7 days (**Figure 4A**). Emodin treatment did not affect the tumor size and weight (**Figure 4B** and **4C**), but significantly reduced macrophage infiltration in the tumors, particularly CD206^+^ M2-like macrophages (**Figure 4D and S8A**) as well as the average cell surface CD206 level of macrophages (**Figure 4E**). Furthermore, emodin treatment increased both CD4^+^ and CD8^+^ T cells in the tumors (**Figure 4F and S8B**); among them, the percentage of IFNγ^+^ cells also was increased in the tumors of emodin-treated mice (**Figure 4G and S8C**). Consistent with our previous findings 16, these data suggest that emodin ameliorated immunosuppression and improved anti-tumor immunity in the TME. In line with the *in vitro* data, emodin treatment significantly decreased CD44^+^/CD24^-^ CSCs in the tumors (**Figure 4H and S8D**) and reduced the expression of EMT markers Vimentin and MMP9 (**Figure 4I**). Correlation analysis indicated positive correlations among the changes in M2-like macrophage infiltration, CSC populations, and EMT markers (**Figure 4J**).

### Emodin suppresses post-surgery breast cancer lung metastasis as a neoadjuvant therapy

**Figure 5:**
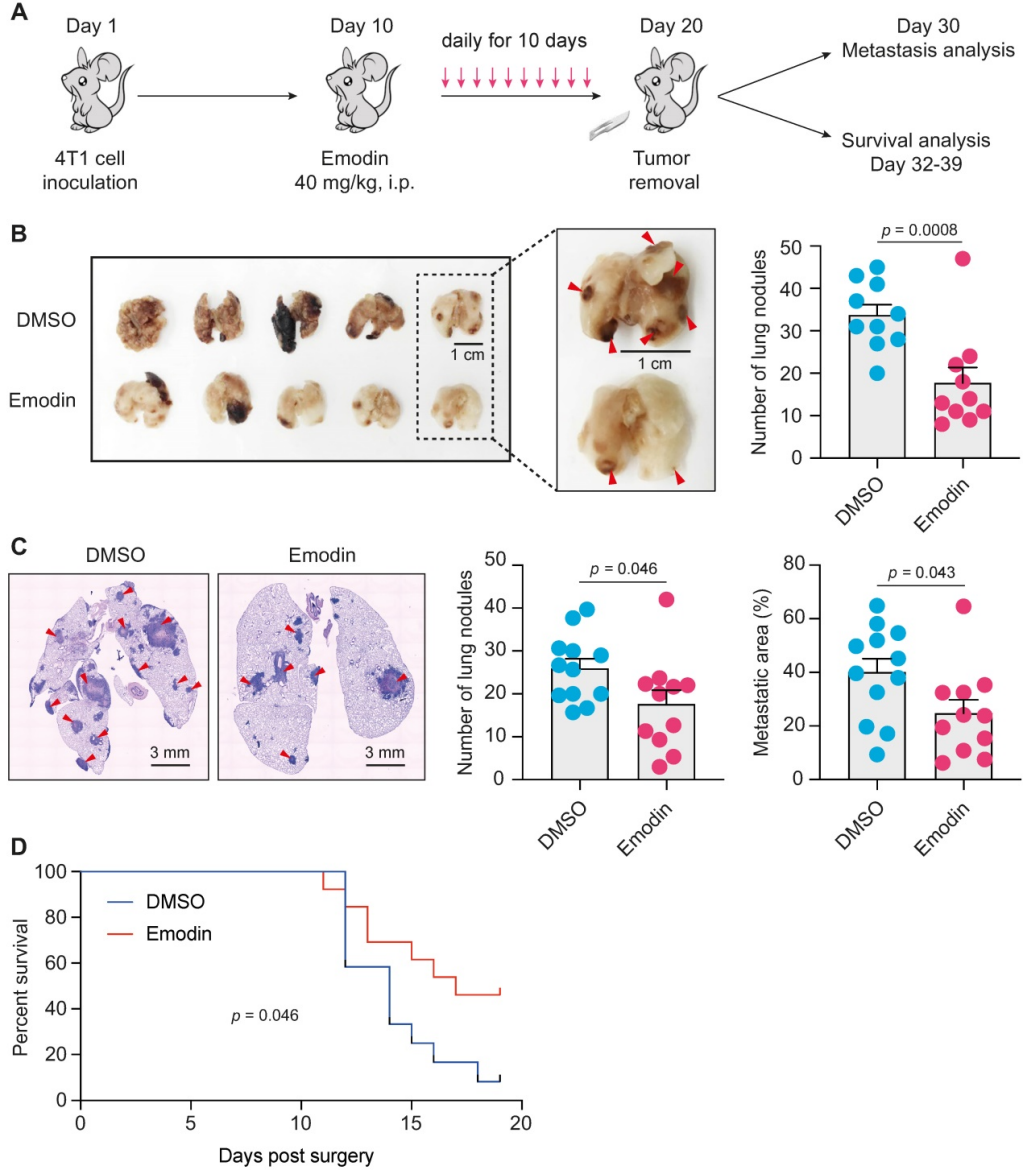
** Emodin suppresses post-surgery breast cancer lung metastasis as a neoadjuvant therapy. A.** Schematic diagram of the design of the experiment. After inoculation of 4T1 cells in mammary fat pads of BALB/C mice, emodin (40 mg/kg) or 1% DMSO in PBS was i.p. injected daily from Day 10 (tumors became palpable) to Day 19. The tumors were completely surgically removed on Day 20. Half of the mice were sacrificed on Day 30 for lung metastasis measurement, and the other half of the mice were used for survival observation till Day 39; **B.** After the mice were sacrificed on Day 30, the lungs were collected for metastasis analysis. Representative images of lung surface metastasis are shown; and the surface nodules of metastasis are quantified;** C**. Lung sections were stained by H&E, and representative images of metastatic nodules inside the lung are shown and quantified; **D.** Overall survival curve of the mice was plotted after Kaplan-Meier analysis.

EMT and TICs play critical roles in cancer metastasis and recurrence 44, 45, and are thus being exploited as therapeutic targets for metastatic breast cancer 14, 46. After acquiring mesenchymal characteristics following EMT, the pioneering tumor cells with stemness properties transendothelially migrate into the intratumoral microvessels and become circulating tumor cells (CTC), and the following extravasation enables these CTCs to disseminate and seed into distant organs to form new tumors 47, 48. CTCs in the peripheral blood behave like TICs, and stem cell and EMT markers are frequently overexpressed in CTCs 48-50. One of the putative reasons for cancer metastatic recurrence is that a small number of cancer cells with TIC properties already exist and are disseminated into circulation when surgery is performed 51. Given the impact of emodin on anti-tumor immunity, EMT and CSCs, we tested if emodin could halt breast cancer post-surgery metastasis as a neoadjuvant therapy (**Figure 5A**). Mice bearing 4T1 tumors were treated with emodin for 10 days prior to complete surgical removal of the tumors. Half of the mice were sacrificed 10 days after the surgery to determine lung metastasis and the other half were further monitored for survival. While the removed tumors were of similar size, emodin substantially reduced lung metastasis on Day 30 (**Figure 5B** and** 5C**). Furthermore, emodin significantly increased the survival compared to the vehicle treated control (**Figure 5D**).

### Emodin inhibits both canonical and noncanonical pathways of TGF-β1 signaling in breast cancer cells

**Figure 6:**
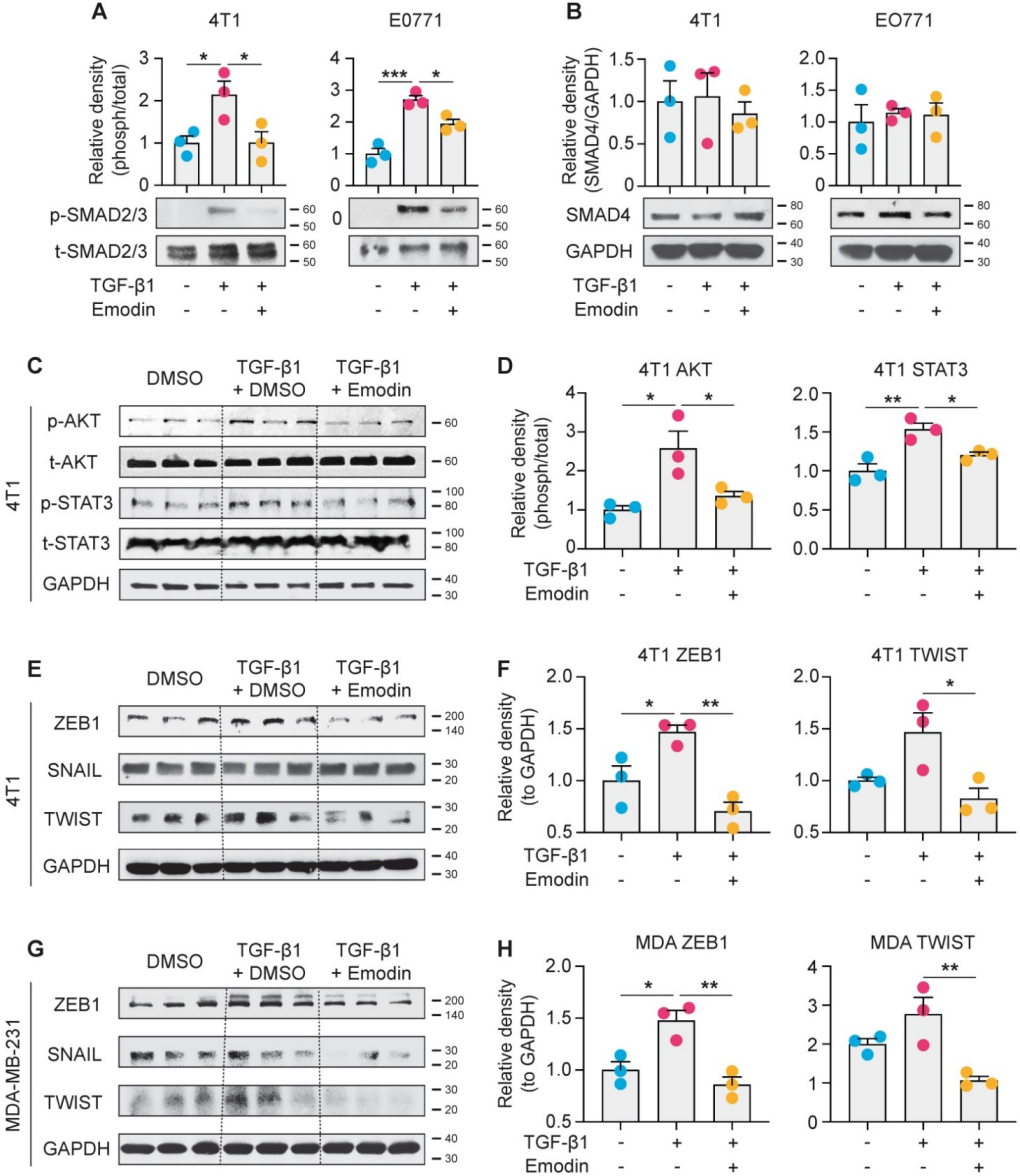
** Emodin suppresses both canonical and noncanonical pathways of TGF-β1 signaling in breast cancer cells. A.** 4T1 and EO771 cells were stimulated by TGF-β1 with or without emodin for 1 h. Western blot was used to detect the phosphorylation and total protein of smad2/3; and the band densities were quantified using three samples in each group;** B.** Total protein of smad4 was detected by western blot and quantified using three samples in each group;** C-D.** The phosphorylation and total protein levels of STAT3 and AKT were detected in 4T1 cells after 1 h treatment as indicated (**C**), and quantifications of the band densities are shown (**D**);** E-H.** The transcription factors for EMT were detected in 4T1 and MDA-MB-231 cells using western blot (**E,G**), and the quantifications of the relative band densities are shown (**F,H**). *P<0.05; **P<0.01.

We next examined the molecular mechanisms by which emodin suppresses TGF-β1-mediated effects on breast cancer cells. The canonical signaling of TGF-β1 is activated through the TGFβRI receptor to induce nuclear translocation and transcriptional activity of Smad proteins, while the noncanonical signaling occurs independently of Smad and involves activation of PI3K-AKT and JAK-STAT3 pathways 27, 52-54. We found that TGF-β1 activated phosphorylation of Smad2/3 and induced Smad2/3 nuclear translocation in breast cancer cells; and both Smad2/3 phosphorylation and nuclear translocation were significantly inhibited by emodin (**Figure 6A and S9**). However, the protein level of Smad4 was not affected (**Figure 6B**). For the noncanonical pathway of TGF-β1 signaling, we tested the inter-connected PI3K-AKT and JAK-STAT3 pathways. We found that the TGF-β1-induced phosphorylation of AKT was inhibited by emodin; in addition, while the phosphorylation of STAT3 was slightly, but significantly, induced by TGF-β1, it was inhibited by emodin (**Figure 6C** and** 6D**). EMT in breast cancer cells is directly regulated by several TFs, such as Zeb1, Snail and Twist 55, and these TFs also are involved in regulating cancer cell stemness 56-58; thus the expression of these TFs was examined. We found that the induction of Zeb1 and Twist by TGF-β1 was observed in both 4T1 cells and MDA-MB-231 cells, and emodin dramatically decreased the induction (**Figures 6E-H**). Taken together, these data suggest that emodin may act on very early steps in TGF-β1/TGFβRI signaling, possibly by directly disrupting TGF-β1/TGFβRI binding.

### TGF-β1 signaling-mediated crosstalk between cancer cells and macrophages has prognostic value

**Figure 7:**
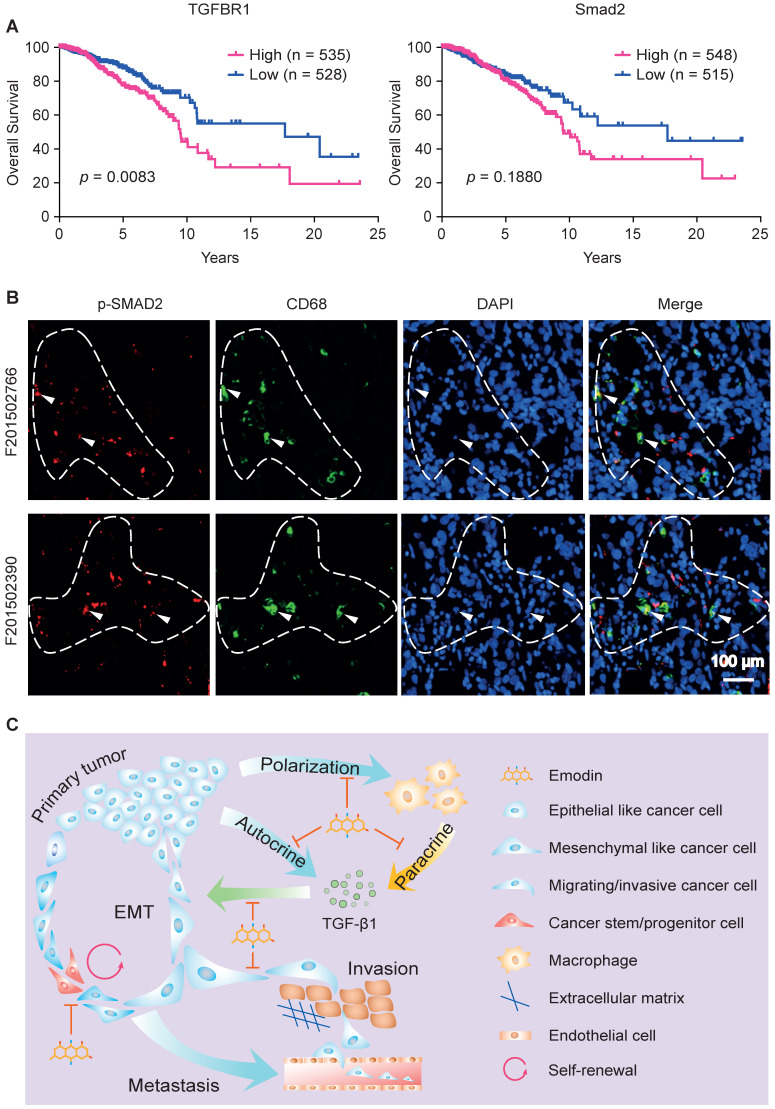
** Clinical implication of TGF-β1 signaling-mediated crosstalk between cancer cells and macrophages. A.** Overall survival curves of invasive breast cancer patients were generated from the TCGA database using a Kaplan-Meier plot. Cutoffs of the high and low expression of the indicated genes were determined by the median values; **B.** Patient samples were used to detect the phosphorylated Smad2 (p-Smad2, red) and CD68 (green) in breast tumor tissues by immunofluorescence staining. DAPI was used to stain nucleus (blue). Arrowheads indicate the colocalization of CD68 and p-Smad2, while dashed lines indicate the proximity of CD68 and Smad2. **C**. Schematic diagram of mechanism of action by which emodin halts metastatic recurrence of breast cancer. TGF-β1 produced by cancer cells and macrophages in the TME promotes EMT of tumor cells via autocrine and paracrine manners. TGF-β1 production in both cancer cells and macrophages is inhibited by emodin. Migration and invasion enable cancer cells to metastasize into distant organs, while stemness and progenitor properties render cancer cells capable of self-renewal during the disseminating and seeding into distant organs. Inhibition of these processes by emodin leads to reduced post-surgery metastatic recurrence of breast cancer.

As TGFβR1 and its alleles were reported to be associated with increased risk of cancers and to contribute to the progression of breast cancer 59, 60, we evaluated the clinical involvement of TGFβR1 in breast cancer prognosis based on the TCGA database. A Kaplan-Meier plot clearly shows that overall survival rate is negatively correlated with TGFβR1 expression level (**Figure 7A, left**). Consistent with this, we observed a similar negative prediction value of Smad2 in the prognosis of breast cancer patients, although not statistically significant (**Figure 7A, right**).

We last examined whether there is a correlation between TGFβR1/Smad2 expression and tumor infiltrated macrophages in human breast tumors. Patient samples were used to detect the phosphorylated Smad2 (the activated form of Smad2) and CD68 (a pan-macrophage marker) in breast tumor tissue using immunofluorescence staining. We observed colocalization of CD68 and p-Smad2, suggesting activated TGF-β1/TGFβR1 signaling in macrophages (**Figure 7B**, marked by arrowheads). Importantly, we noticed that p-Smad2 also is present in the nearby CD68^-^ cancer cells (**Figure 7B**, within dash line). These results indicate that TGF-β1/TGFβR1 signaling is activated in both macrophages and tumor cells, and their spatial proximity suggests a paracrine interaction between macrophages and tumor cells through TGF-β1/TGFβR1 signaling. These data imply that emodin shall also be effective in halting breast cancer metastatic recurrence in humans through blocking TGF-β1/TGFβR1 signaling-mediated crosstalk between cancer cells and macrophages.

## Discussion

Emodin has been reported to harbor therapeutic potential for several diseases 61. Its effectiveness in cancer has largely been shown in cell culture studies that suggest that emodin directly promotes apoptosis or inhibits proliferation of cancer cells 62. Our published studies showed that emodin inhibited breast cancer growth and metastasis in orthotopic mouse models 16, 17. We found that, at concentrations achievable *in vivo*, emodin exerted anti-breast cancer effects not through directly killing cancer cells, but rather through reducing macrophage recruitment to tumors and lungs and suppressing their M2-like polarization via actions on both macrophages and breast cancer cells 16, 17. In this current study, we expanded on our initial findings to demonstrate that emodin suppresses breast cancer cell EMT and reduces CSC/TIC numbers, through blocking the TGF-β1-mediated interaction between macrophages and breast cancer cells (**Figure 7C**). Moreover, using a mouse model, we show for the first time that emodin, as a neoadjuvant monotherapy, can effectively reduce breast cancer post-surgery metastatic recurrence in the lungs.

Myeloid cells, especially macrophages, are abundant in the TME of many solid tumors, including breast cancer, and contribute to cancer metastasis 63. In addition to causing immune suppression, TAMs also promote cancer cell EMT by secreting a plethora of cytokines, chemokines and growth factors, particularly TGF-β1 18-20. By analyzing the TCGA database, we confirmed that macrophage abundance is positively correlated with TGF-β1 expression, and EMT and CSC markers in breast tumors (**Figure 1**). While TGF-β1 functions as a tumor suppressor during the early stage of tumorigenesis, as tumors progress, tumor cells lose their growth-inhibitory response to TGF-β1 and instead respond to TGF-β1 to undergo EMT and acquire capability of migration and invasion 8. Using a TGF-β1-induced EMT cell culture model, and direct or indirect macrophage-breast cancer cell co-culture models, we show that emodin suppresses both TGF-β1-induced and macrophage-triggered breast cancer cell EMT, and thus breast cancer cell migration and invasion (**Figure 2**). Of note, breast cancer cells can respond to their own TGF-β1 (autocrine effect) and TGF-β secreted from macrophages (paracrine effect) to undergo EMT. We demonstrated that emodin not only suppressed the cancer cell response to TGF-β1, but also inhibited TGF-β1 expression in both macrophages and cancer cells (**Figure 2**).

While the inhibitory effects of emodin on macrophage production of TGF-β1 can be explained by its suppression of M2-like macrophage polarization as we previously elucidated 16, 17, the mechanism by which emodin reduces TGF-β1 production in breast cancer cells and inhibits TGF-β1-induced breast cancer cell EMT likely involves blockage of TGFβR1 signaling. Our data show that emodin suppresses both canonical and noncanonical pathways of TGF-β1 signaling (**Figure 6**), suggesting that emodin interferes the early events in TGF-β1/TGFβRI signaling likely by blocking TGF-β1/TGFβRI binding. Several direct targets of emodin have been identified, including protein tyrosine kinase p65lck 64, casein kinase 2 (CK2) 65, and β-hydroxyacyl-acyl carrier protein dehydratase 66; among them, CK2 is the best characterized one; emodin binds to it with high affinity 65, 67. However, inhibition of these proteins cannot fully explain the effects of emodin we observed in this study.

The EMT process generates cells with properties of stem cells 13. TICs, including CSCs and cancer progenitor cells, have emerged as promising targets for cancer therapy due to their roles in cancer metastasis and therapy-resistance 68. Therefore, we examined whether the EMT-facilitated TIC population generation and maintenance could be inhibited by emodin. Indeed, we found that emodin could effectively reduce TGF-β1-induced expression of CSC markers in multiple breast cancer cell lines and diminish their ability to form mammospheres *in vitro* (mammosphere formation assays) and to generate tumors *in vivo* (limited dilution assays) (**Figure 3**). We further demonstrated that emodin suppressed TGF-β1-induced expression of several transcription factors that are critical to CSC generation and maintenance, such as FoxC2, Nanog, Oct4, Jagged1, and KLF4 (**Figure 3**).

The major significance of this study lies in the finding that emodin substantially halts post-surgery breast cancer metastasis in mouse models (**Figure 4** and** 5, Figure S8**). First, we showed that 7 daily emodin treatments in mice with established orthotopic breast tumors alleviated tumor immunosuppression of the TME, improved anti-tumor immunity, and suppressed EMT and CSCs independent of any change in tumor size (**Figure 4**), demonstrating that the identified *in vitro* actions of emodin all operate *in vivo*. Second, we showed that emodin as a neoadjuvant therapy (10 daily treatments in mice with established orthotopic breast tumors prior to surgical removal of the tumors) significantly reduced post-surgery breast cancer lung metastasis and improved mouse survival (**Figure 5**). These experiments demonstrated that emodin, as a neoadjuvant therapy can significantly reduce breast cancer post-surgery metastasis.

The effectiveness of emodin in blocking the TGF-β1-mediated crosstalk between macrophages and breast cancer cells has evident clinical implications. As we showed, analysis of the TCGA database revealed a prognostic role of TGFβR1 and p-Smad2 in the overall survival of breast cancer patients, and examination of breast cancer tissue samples indicated the proximity of macrophage marker CD68 and p-Smad2 in breast cancer cells (**Figure 7**). One may argue that specific TGF-β1 signaling inhibitors such as SB431542 could be better choices than emodin in clinical applications. However, clinical use of TGF-β1 signaling inhibitors has been hindered by their severe side effects due to the many vital physiological roles of TGF-β1. We believe that the natural compound emodin has intrinsic advantages compared to those specific TGF-β1 signaling inhibitors in that 1) with the relatively mild nature of its TGF-β1 signaling inhibition, emodin may only suppress excessive TGF-β1 signaling in the tumors while sparing its physiological activities; and 2) in addition to suppression of TGF-β1 signaling, emodin also acts on macrophage-breast cancer cell interactions through other mechanisms as we showed previously 16, 17.

In summary, while more work is still needed to confirm if emodin indeed directly blocks the interaction between TGF-β1 and TGFβR1, this study provides convincing pre-clinical evidence suggesting that emodin harbors the potential for clinical development as a new effective and safe agent to halt metastatic recurrence of breast cancer, either as a monotherapy, or in combination with other neoadjuvant or adjuvant therapies.

## Supplementary Material

Supplementary figures and table.

## Abbreviations

ALDH: aldehyde dehydrogenase activity
CSC: cancer stem cells
CTC: circulating tumor cell
EMT: epithelial-mesenchymal transition
ERK: extracellular signal regulated kinase
MAPK: mitogen-activated protein kinase
MET: mesenchymal-epithelial transition
MFI: median fluorescent intensity
MMTV: mouse mammary tumor virus
PC: progenitor cells
PI3K: phosphoinositide 3-kinase
PMCM: peritoneal macrophage conditioned medium
PyMT: Polyoma virus middle T antigen
STAT: signal transducer and activator of transcription
TAM: tumor-associated macrophages
TCCM: tumor cell conditioned medium
TF: transcription factor
TGF-β: transforming growth factor-β
TIC: tumor initiating cells
TNBC: triple-negative breast cancer
TβRI/II: TGF-β receptor type I/II
