# Supplementary material for: Emodin reduces Breast Cancer Lung Metastasis by suppressing Macrophage-induced Breast Cancer Cell Epithelial-mesenchymal transition and Cancer Stem Cell formation
Source: Theranostics. 2020 Jul 9;10(18):8365–81. doi: 10.7150/thno.45395 (PMC7381725; doi:10.7150/thno.45395)
Supplement: Supplementary file 1 — Supplementary figures and table. [file thnov10p8365s1.pdf]

Table S1: The sequences of primers used in this study

Primers		Sequences (5'-3')	Primers		Sequences (5'-3')
**mNcad**	**F**	GGGACAGGAACACTGCAAAT	**hVim**	**F**	CTTCGTGAATACCAAGAC
	**R**	CGGTTGATGGTCCAGTTTCT		**R**	GATAACCTGTCCATCTCTA
**mVim**	**F**	ATGCTTCTCTGGCACGTCTT	**hFn**	**F**	AGTGGCAGAAGGAATATC
	**R**	AGCCACGCTTTCATACTGCT		**R**	GAGAATACTGGTTGTAGGA
**mFoxC2**	**F**	CACTCTGAACGGCATCTAC	**hMMP2**	**F**	GATGATGTTAGGCAAGTG
	**R**	GGCACATCCTTCTTCTTGA		**R**	GAAGGTGTTCAGGTATTG
**mOct4**	**F**	TCACTCACATCGCCAATC	**hFoxC2**	**F**	TGAGCGAGCAGAATTACTAC
	**R**	CCTGTAGCCTCATACTCTTC		**R**	AGCGGTCCATGATGAACT
**mJagged1**	**F**	GACAACACCACCAACAATG	**hOct4**	**F**	CGCCGTATGAGTTCTGTG
	**R**	TCATCCTCCTCCACTTCC		**R**	GCTCCAGCTTCTCCTTCT
**mNanog**	**F**	GAAGATGCGGACTGTGTT	**hNotch1**	**F**	TCTACCTGGAGATTGACAAC
	**R**	CAGGTTCAGAATGGAGGAG		**R**	ACGAAGAACAGAAGCACAA
**mNotch1**	**F**	AAGTGGACATTGACGAGTG	**hJagged1**	**F**	GTCTTACTACGGAGCACAT
	**R**	TGAGGCATAAGCAGAGGTA		**R**	CGCCTCTGAACTCTTACTT
**mCortactin**	**F**	GCCACTATCAAGCAGAGG	**hNanog**	**F**	TCTCCAACATCCTGAACCT
	**R**	CCAGCCATCGTCAATCAT		**R**	GCGTCACACCATTGCTAT
**mTks5**	**F**	AGGAGGCTGAGGAGAATC	**hKLF4**	**F**	GTCTTGAGGAAGTGCTGAG
	**R**	ATGCTTGTCACCTGAACC		**R**	GGACTGACCTTGGTAATGG
**hNcad**	**F**	CATCATCCTGCTTATCCT	**hGAPDH**	**F**	GAGTCAACGGATTTGGTCGT
	**R**	TAGTCCTGGTCTTCTTCT		**R**	TTGATTTTGGAGGGATCTCG

Supplemental Figures

Figure S1

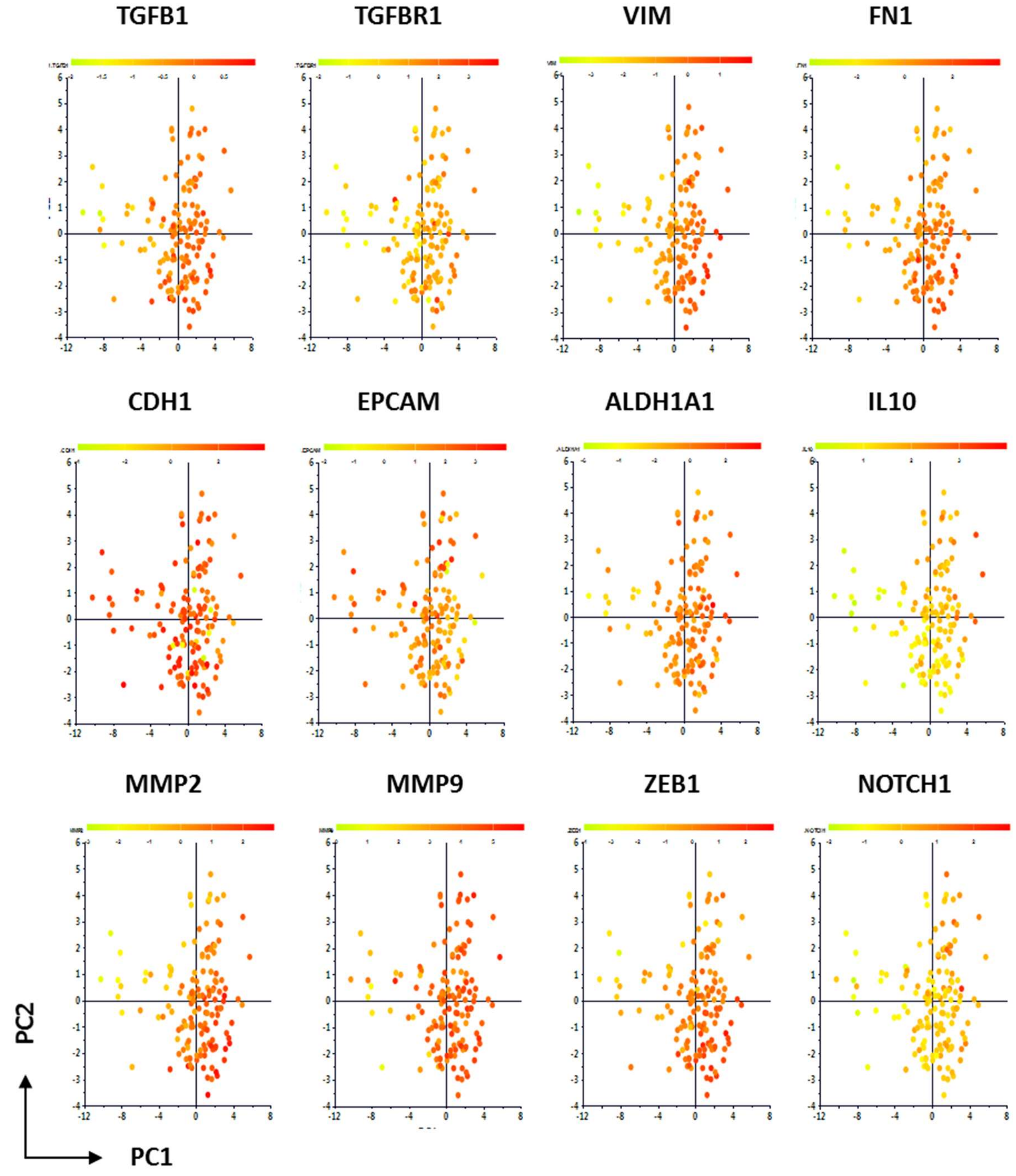

Figure S1. Expression patterns of selected EMT and CSC-related genes in the PCA-categorized groups from Figure 1A. N = 147.

Figure S2

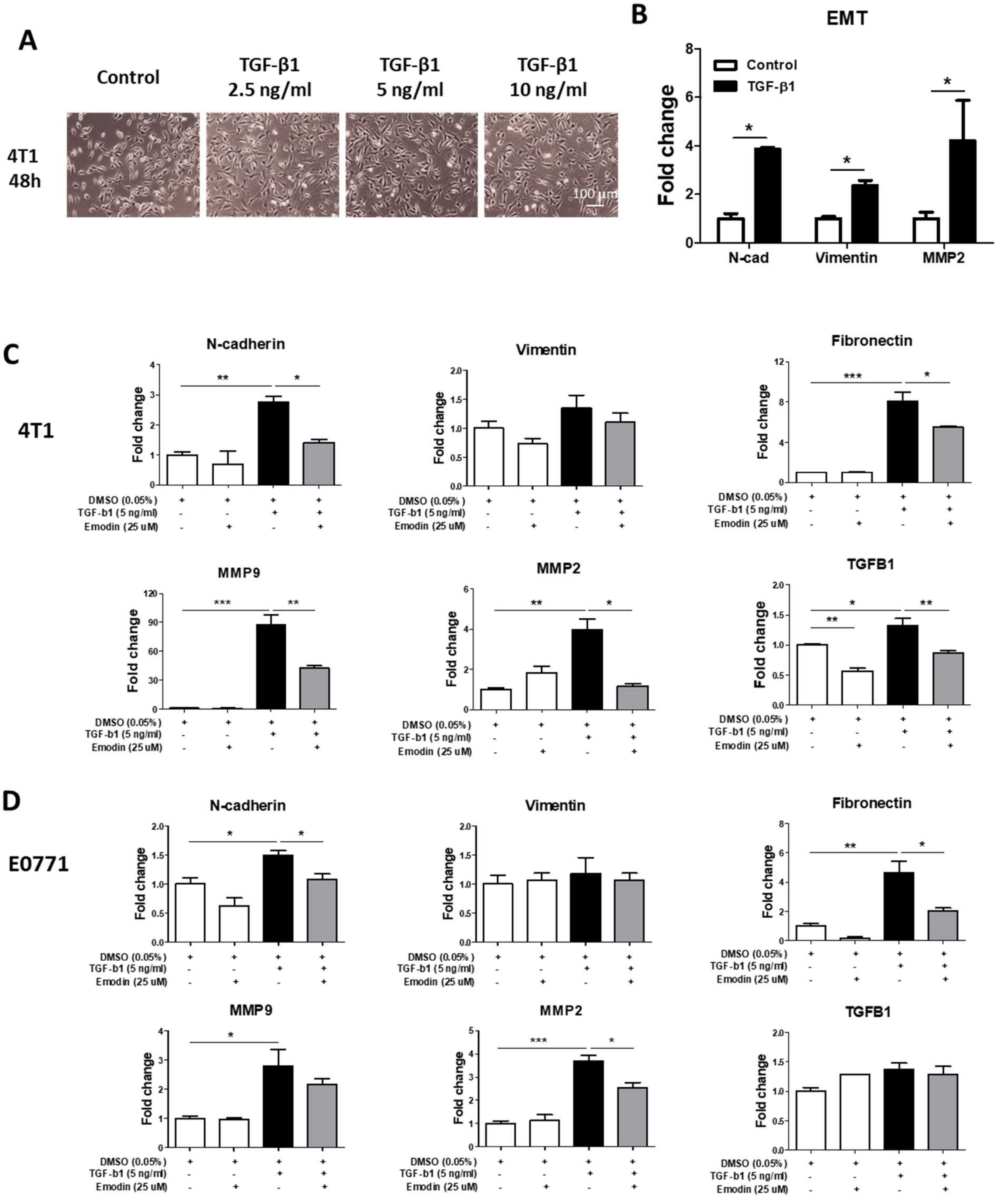
A
Control
TGF-β1 2.5 ng/ml
TGF-β1 5 ng/ml
TGF-β1 10 ng/ml
4T1 48h
100 μm
B
EMT
Control
TGF-β1
Fold change
N-cad
Vimentin
MMP2
C
4T1
N-cadherin
Vimentin
Fibronectin
MMP9
MMP2
TGFB1
Fold change
DMSO (0.05%)
TGF-b1 (5 ng/ml)
Emodin (25 uM)
D
E0771
N-cadherin
Vimentin
Fibronectin
MMP9
MMP2
TGFB1
Fold change
DMSO (0.05%)
TGF-b1 (5 ng/ml)
Emodin (25 uM)

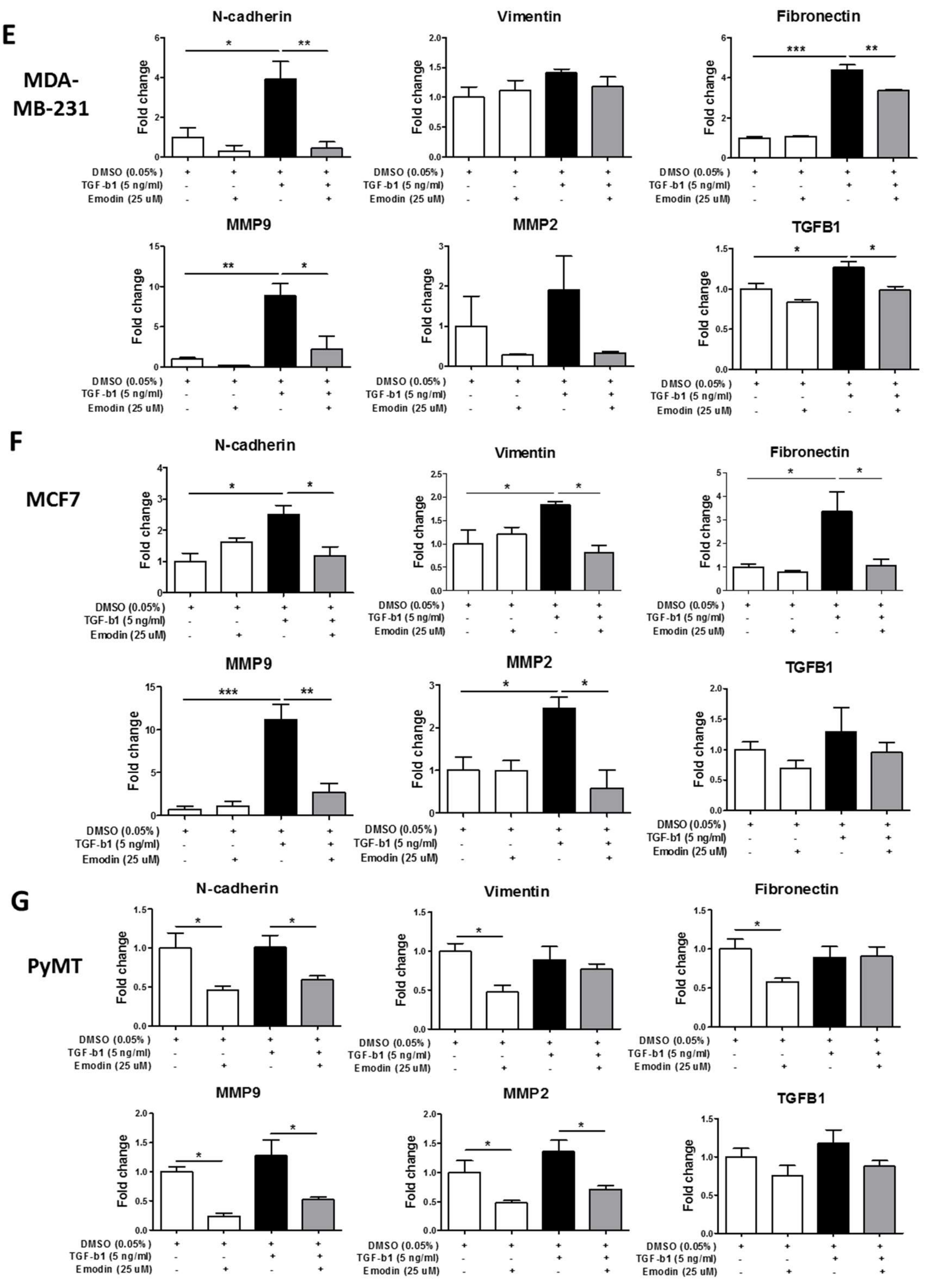
E
MDA-MB-231
N-cadherin
Vimentin
Fibronectin
MMP9
MMP2
TGFB1
F
MCF7
N-cadherin
Vimentin
Fibronectin
MMP9
MMP2
TGFB1
G
PyMT
N-cadherin
Vimentin
Fibronectin
MMP9
MMP2
TGFB1
Fold change
DMSO (0.05%)
TGF-b1 (5 ng/ml)
Emodin (25 uM)

Figure S2. Emodin suppresses TGF-β1-induced EMT of breast cancer cells

A. Typical change of morphology of 4T1 cells under the stimulation of increasing concentrations of TGF-β1. Images show that 4T1 cells acquire fibroblast-like, mesenchymal morphology under TGF-β1 stimulation (original magnification: 100X);

B. qPCR analysis of EMT gene expression in 4T1 cells after TGF-β1 stimulation;

C-G. qPCR analysis of EMT gene expression in various mouse and human breast cancer cell lines and tumor cells isolated from tumors of PyMT mice. The cells were treated with or without TGF-β1 and emodin.

Figure S3

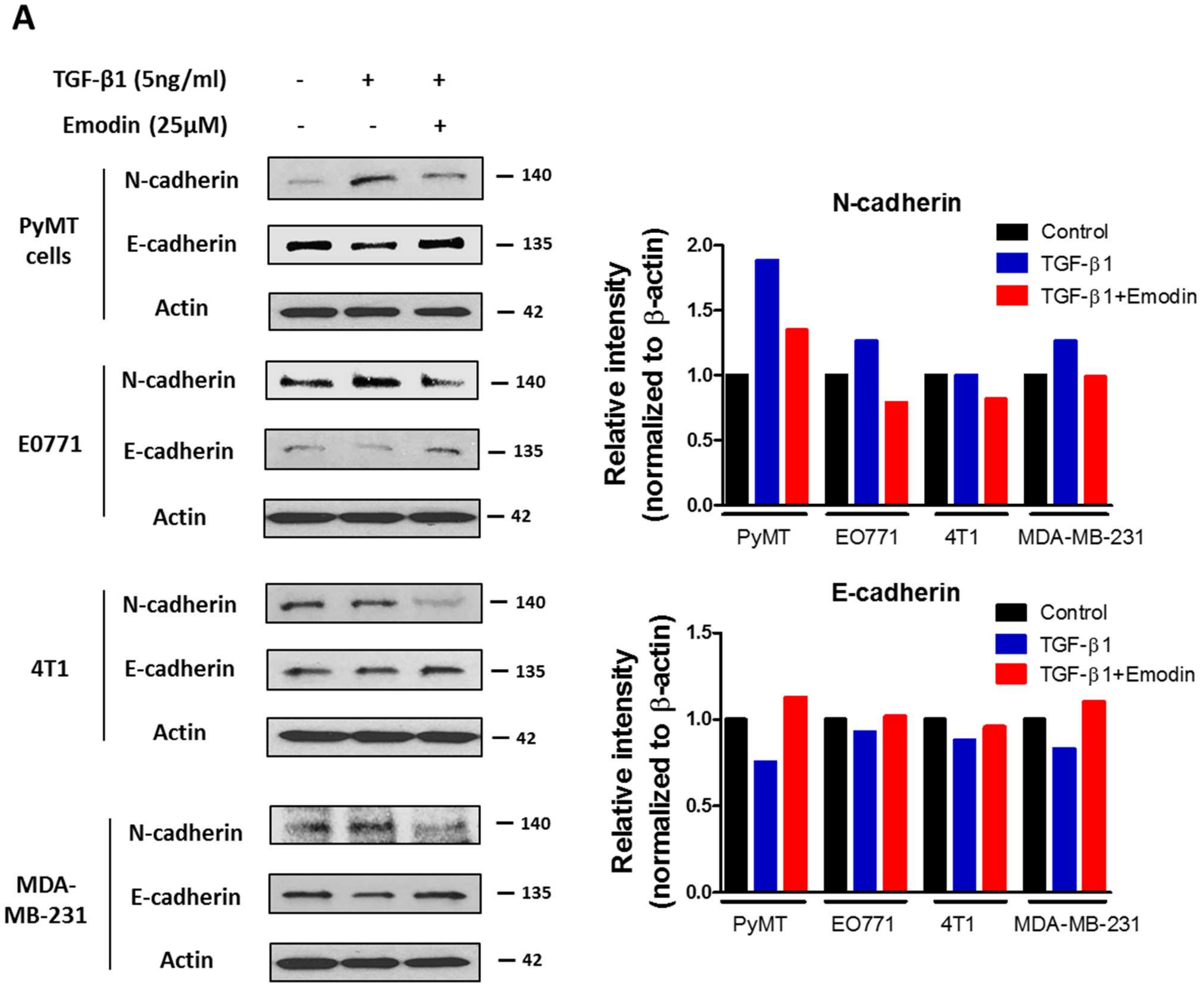

A
TGF-β1 (5ng/ml) - + +
Emodin (25μM) - - +
PyMT cells
E0771
4T1
MDA-MB-231
N-cadherin
E-cadherin
Actin
140
135
42
N-cadherin
E-cadherin
Relative intensity (normalized to β-actin)
Control
TGF-β1
TGF-β1+Emodin
PyMT
EO771
4T1
MDA-MB-231
0.0
0.5
1.0
1.5
2.0

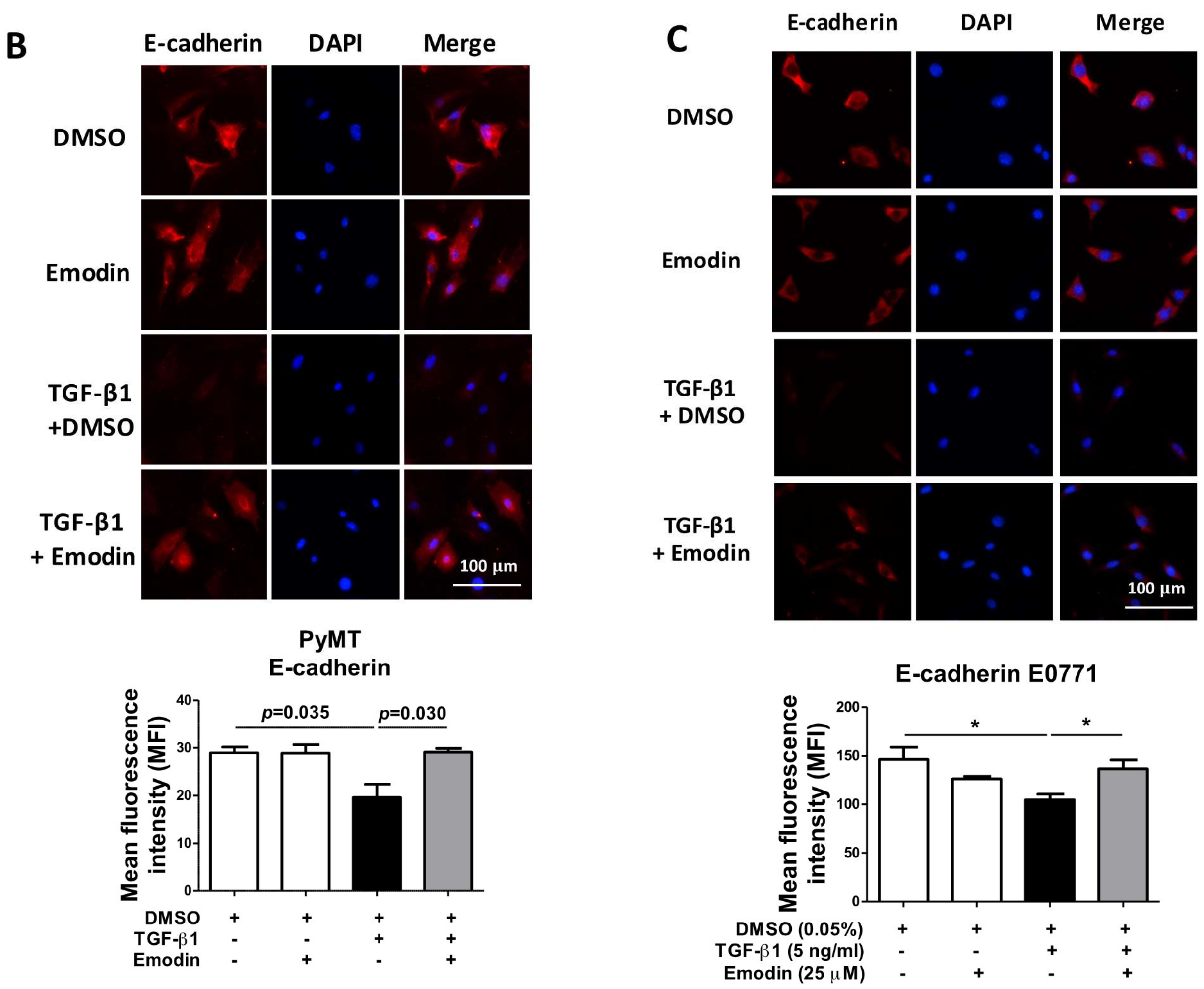

Figure S3. Emodin reverses TGF-β1-induced changes of N-cadherin and E-cadherin protein levels in breast cancer cells

A. Western blot detection of EMT markers N-cadherin and E-cadherin in various breast cancer cells after TGF-β1 stimulation with or without Emodin treatment (48 h). The band intensities were normalized to corresponding β-actin.

B-C. Representative images showing the immunofluorescence staining for E-cadherin in PyMT tumor cells (**B**) and EO771 cells (**C**) following TGF-β1 stimulation with or without Emodin treatment (48 h), and the fluorescence intensities of E-cadherin were quantified using the software IPP. *$P < 0.05$.

Figure S4

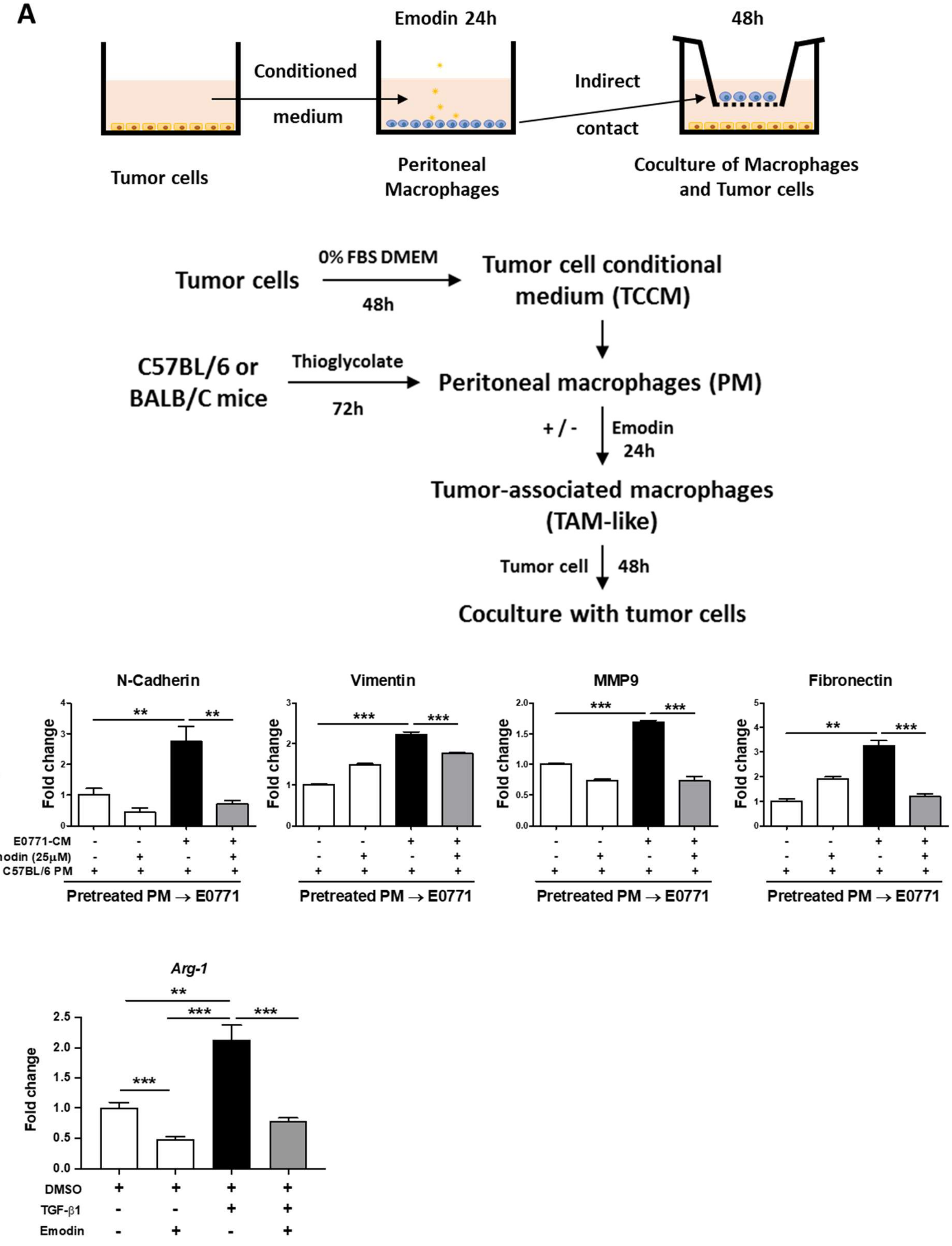
A
Emodin 24h
48h
Conditioned
medium
Indirect
contact
Tumor cells
Peritoneal
Macrophages
Coculture of Macrophages
and Tumor cells
Tumor cells
0% FBS DMEM
48h
Tumor cell conditional
medium (TCCM)
C57BL/6 or
BALB/C mice
Thioglycolate
72h
Peritoneal macrophages (PM)
+ / -
Emodin
24h
Tumor-associated macrophages
(TAM-like)
Tumor cell
48h
Coculture with tumor cells
B
E0771
N-Cadherin
Vimentin
MMP9
Fibronectin
Fold change
E0771-CM
Emodin (25μM)
C57BL/6 PM
Pretreated PM → E0771
C
Arg-1
Fold change
DMSO
TGF-β1
Emodin

Figure S4. Emodin suppresses EMT of breast cancer cells and M2 polarization of macrophages.

A. Schematic diagram for the induction of TAM-like macrophages (TAMs) by treating peritoneal macrophages with tumor conditional medium (TCCM), and the indirect contact coculture of TAMs and breast cancer cells;

B. Emodin inhibits EMT gene expression in coculture of TAMs and EO771 cells;

C. Emodin inhibits Arg-1 expression in peritoneal macrophages with or without TGF-β1 treatment (18 h); n=3; $^{**}p<0.01$; $^{***}p<0.001$.

Figure S5

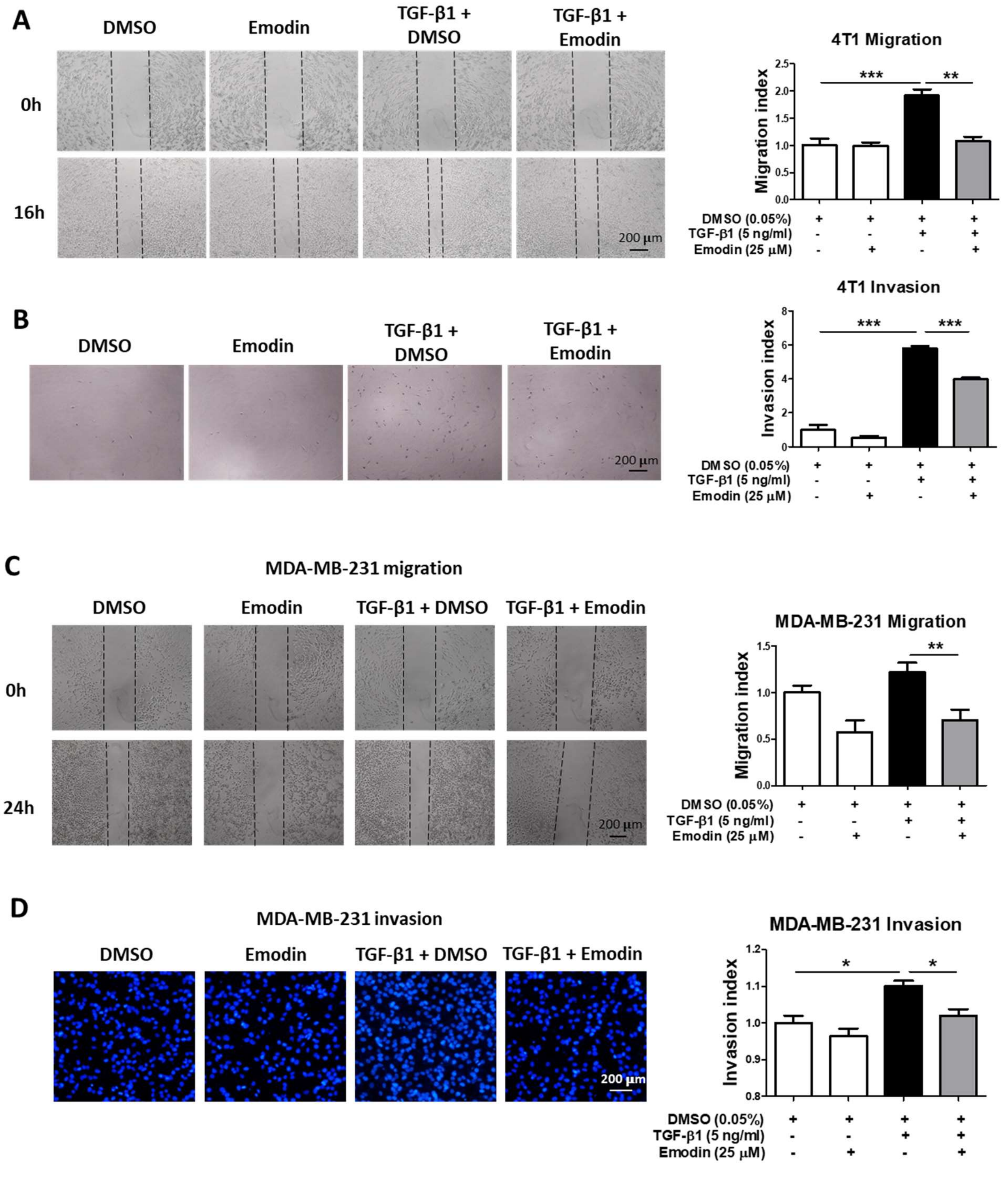
A
DMSO
Emodin
TGF-β1 + DMSO
TGF-β1 + Emodin
0h
16h
200 μm
4T1 Migration
Migration index
DMSO (0.05%)
TGF-β1 (5 ng/ml)
Emodin (25 μM)
B
DMSO
Emodin
TGF-β1 + DMSO
TGF-β1 + Emodin
200 μm
4T1 Invasion
Invasion index
DMSO (0.05%)
TGF-β1 (5 ng/ml)
Emodin (25 μM)
C
MDA-MB-231 migration
DMSO
Emodin
TGF-β1 + DMSO
TGF-β1 + Emodin
0h
24h
200 μm
MDA-MB-231 Migration
Migration index
DMSO (0.05%)
TGF-β1 (5 ng/ml)
Emodin (25 μM)
D
MDA-MB-231 invasion
DMSO
Emodin
TGF-β1 + DMSO
TGF-β1 + Emodin
200 μm
MDA-MB-231 Invasion
Invasion index
DMSO (0.05%)
TGF-β1 (5 ng/ml)
Emodin (25 μM)

E

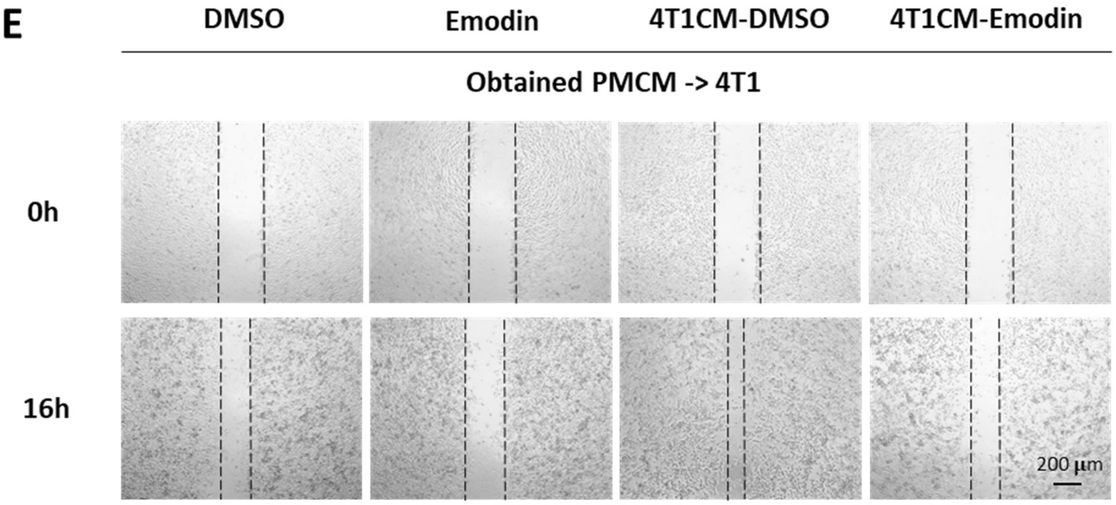
DMSO
Emodin
4T1CM-DMSO
4T1CM-Emodin
Obtained PMCM -> 4T1
0h
16h
200 μm

F

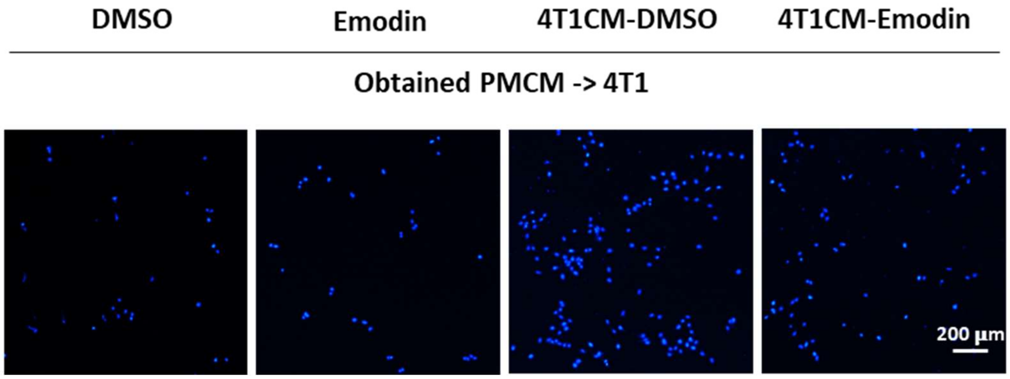
DMSO
Emodin
4T1CM-DMSO
4T1CM-Emodin
Obtained PMCM -> 4T1
200 μm

Figure S5. Emodin suppresses breast cancer cell migration and invasion

A. Wound-healing assay of 4T1 cell migration following TGF-β1 stimulation with or without emodin treatment (16 h); representative images and quantification are shown; Migration index = (Width before migration–Width after migration)/Width before migration, all data were normalized to the first group; **P*<0.05; ***P*<0.01;

B. Matrigel invasion assay of 4T1 cells following TGF-β1 stimulation with or without emodin treatment; quantification of the invasion index of 4T1 cells by the software IPP. Matrigel concentration: 300 ug/ml, Transwell pore size: 8 uM, Invasion time: 24 h, Invasion index is calculated by the normalization of cell number per field in each group to the DMSO control group;

C. Wound-healing assay of the MDA-MB-231 cell migration following TGF-β1 stimulation with or without emodin treatment. Representative images and quantification are shown;

D. Matrigel invasion assay of the invasiveness of MDA-MB-231 cells following TGF-β1 stimulation with or without emodin treatment; and representative images of the invaded cells into the bottom wells stained with DAPI and quantification are shown.

E. Wound-healing assay of the 4T1 cell migration following PMCM stimulation with or without emodin treatment (16 h). Representative images are shown. Quantification is shown in Figure 2F.

F. Matrigel invasion assay of 4T1 cells following PMCM stimulation with or without emodin treatment. Representative images of the invade cells staining with DAPI are shown. Quantification is shown in Figure 2G.

Figure S6.

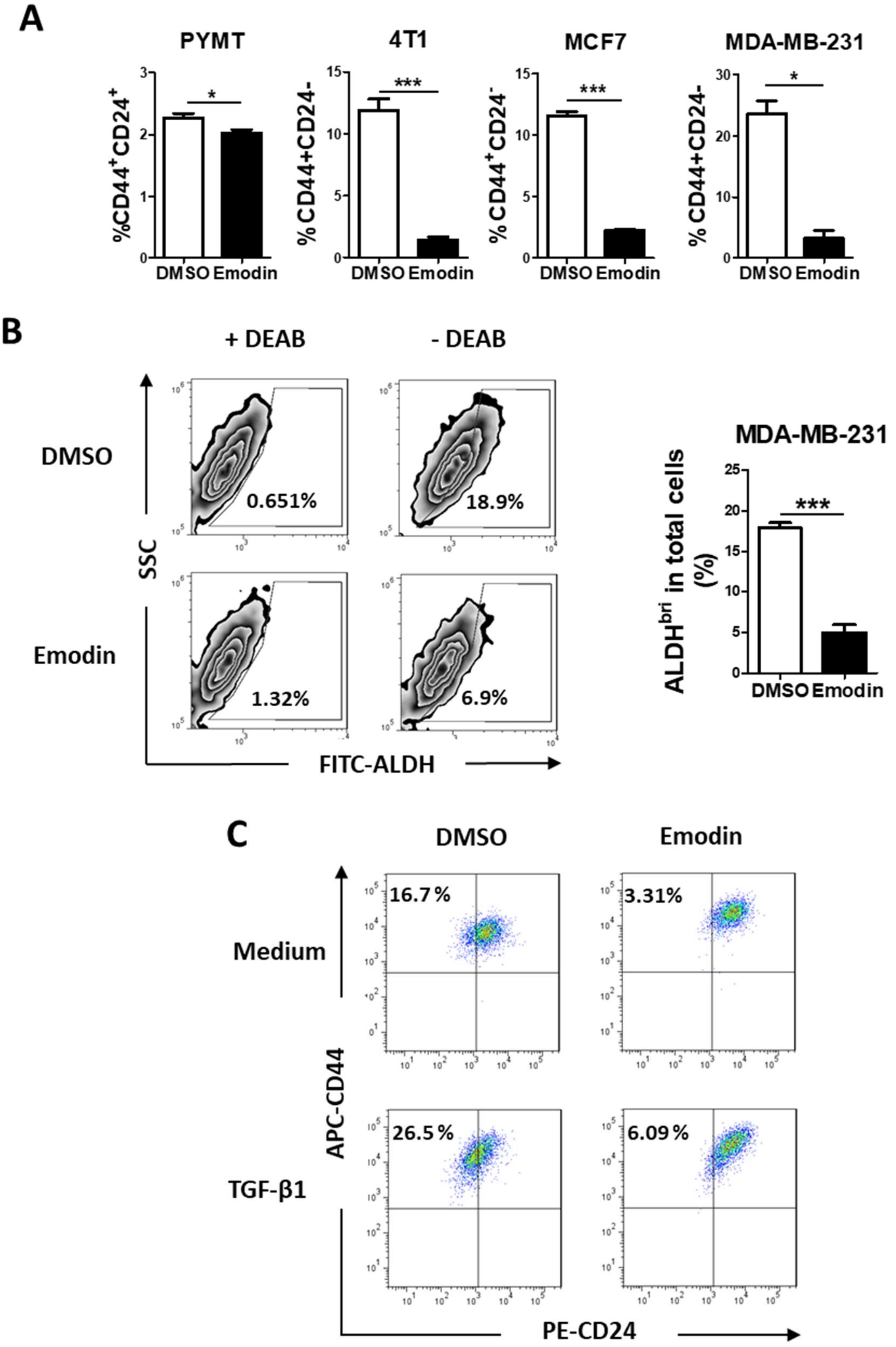
A
PYMT
4T1
MCF7
MDA-MB-231
%CD44+CD24+
% CD44+CD24-
% CD44+CD24-
% CD44+CD24-
DMSO Emodin
*
***
***
*
B
+ DEAB
- DEAB
DMSO
Emodin
0.651%
18.9%
1.32%
6.9%
SSC
FITC-ALDH
MDA-MB-231
ALDHbri in total cells (%)
***
DMSO Emodin
C
DMSO
Emodin
Medium
TGF-β1
16.7%
3.31%
26.5%
6.09%
APC-CD44
PE-CD24

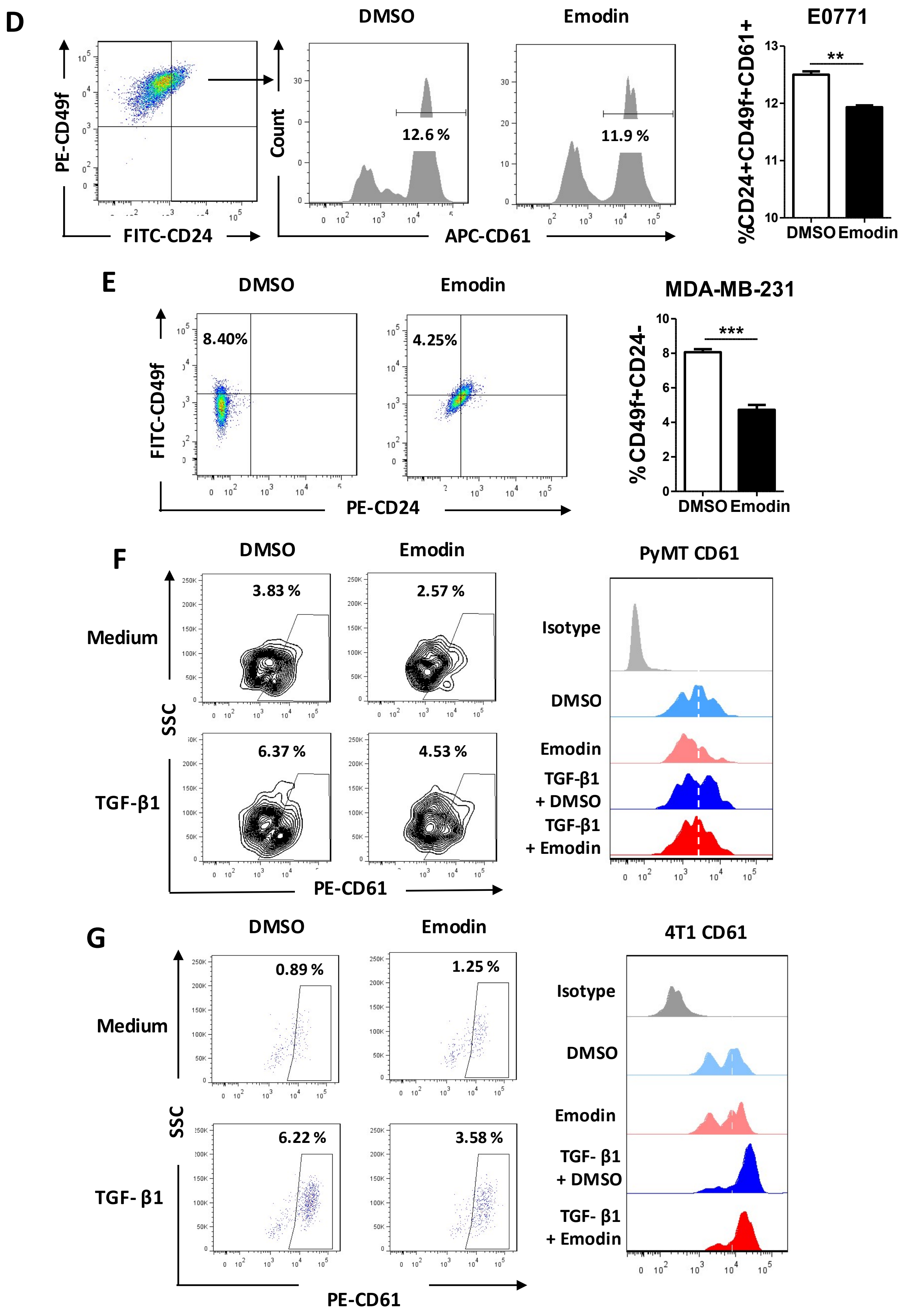
D
DMSO
Emodin
E0771
PE-CD49f
FITC-CD24
Count
12.6 %
11.9 %
APC-CD61
%CD24+CD49f+CD61+
**
DMSO Emodin
E
DMSO
Emodin
MDA-MB-231
8.40%
4.25%
FITC-CD49f
PE-CD24
% CD49f+CD24-
***
DMSO Emodin
F
DMSO
Emodin
PyMT CD61
3.83 %
2.57 %
Medium
6.37 %
4.53 %
TGF-β1
SSC
PE-CD61
Isotype
DMSO
Emodin
TGF-β1
+ DMSO
TGF-β1
+ Emodin
G
DMSO
Emodin
4T1 CD61
0.89 %
1.25 %
Medium
6.22 %
3.58 %
TGF- β1
SSC
PE-CD61
Isotype
DMSO
Emodin
TGF- β1
+ DMSO
TGF- β1
+ Emodin

Figure S6. Emodin reduces progenitor cell population in breast cancer cells

A. Flow cytometry analysis of the effect of emodin on cancer stem cell populations in various breast cancer cells (24 h). The quantification of cancer stem cell percentages is shown;

B. ALDEFLUOR assay of the inhibitory effects of Emodin on cancer stem cell population ($ALDH^{bri}$) in MDA-MB-231 cells (24 h). The representative flow cytometry graphs and the quantification of cancer stem cell percentages are shown;

C. Flow cytometry analysis of the effect of emodin on cancer stem cell populations in 4T1 cells with or without TGF-β1 stimulation (24 h). The representative images are shown;

D-E. Effects of emodin on progenitor cell populations in EO771 cells (**D**) and MDA-MB-231 cells (**E**) were analyzed by flow cytometry. The representative flow cytometry graphs and quantification of progenitor cell percentages are shown;

F-G. Flow cytometry analysis of the effect of emodin on progenitor cell populations in PyMT (**F**) and 4T1 (**G**) cells after TGF-β1 stimulation for 48 h; the representative flow cytometry images of the progenitor cell percentages and the mean fluorescence intensity of CD61 are shown. The CD61+ subpopulation was gated from the CD24+CD49f+ cell population.

Figure S7.

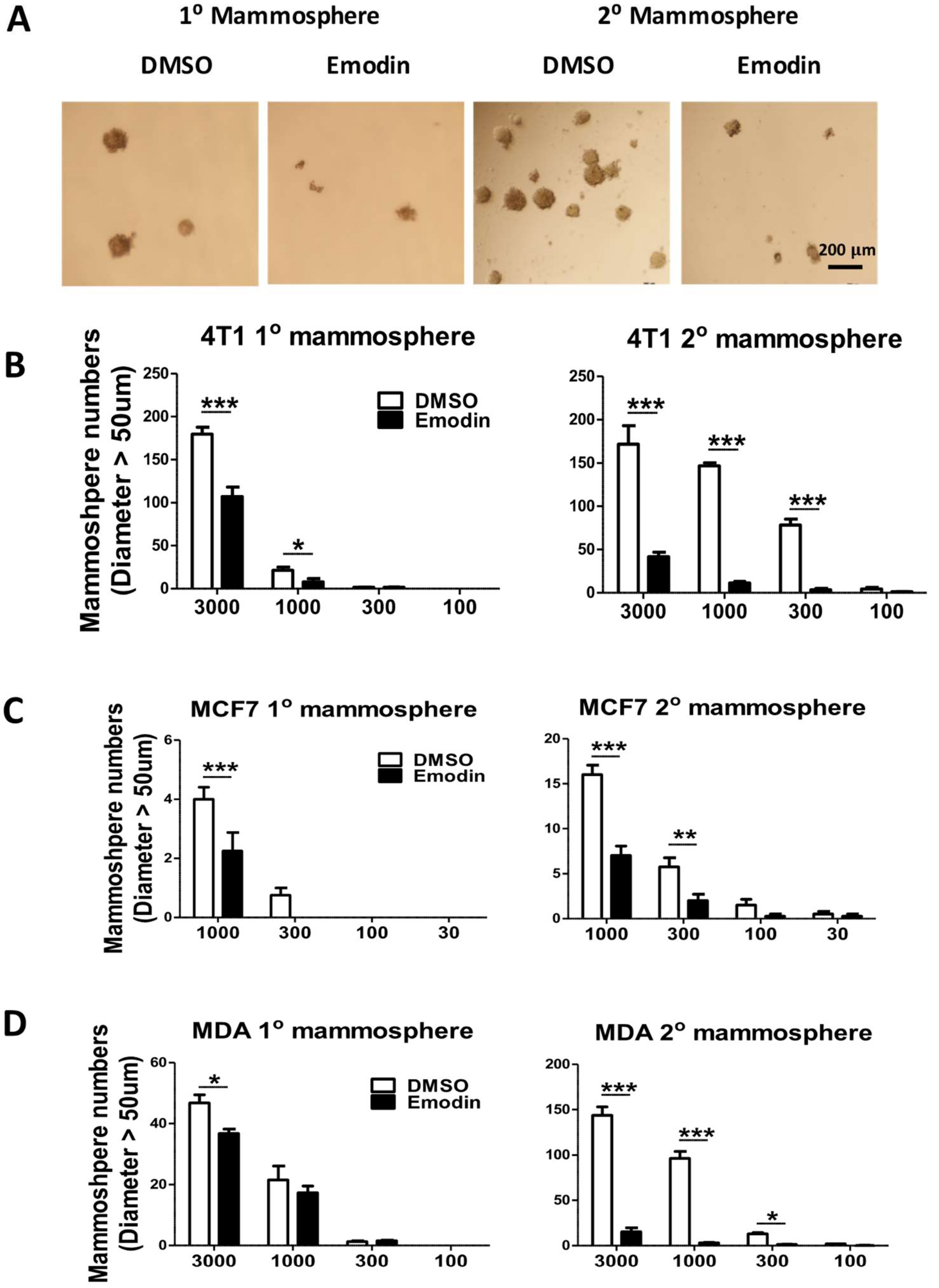

E

MDA-MB-231 injected	Tumor incidence/Injections in mice				Estimated stem cell frequency (with 95% Confidence intervals)	
	$1X10^5$	$4X10^4$	$2X10^4$	$1X10^4$		
**DMSO**	5/5	3/5	2/5	0/5	1 in 41760	(21454 - 81284)
**Emodin**	3/5	1/5	0/5	1/5	1 in 131004	(54156 - 316904) *

* p=0.0402 vs DMSO group.

Figure S7. Emodin suppresses mammosphere formation and tumor formation from breast cancer cells

A. Representative images show that emodin inhibits tumor mammosphere formation in EO771 cells. The 1st mammospheres were cultured in ultra-low attachment plates for 7 days, and the 2nd mammospheres were cultured for another 7 days;

B-D. Quantification of the number of formed 1st mammospheres and 2nd mammospheres in various breast cancer cells (**B**. 4T1; **C**, MCF7; and **D**, MDA-MB-231) with or without emodin treatment;

E. Limiting dilution assay shows the inhibitory effect of emodin on breast cancer stem cells in MDA-MB-231 cells. The data are presented as the number of mice with detectable tumors (0-5) versus total number of mice injected with cancer cells (5). The estimated stem cell frequency was calculated for statistical analysis (right).

Figure S8.

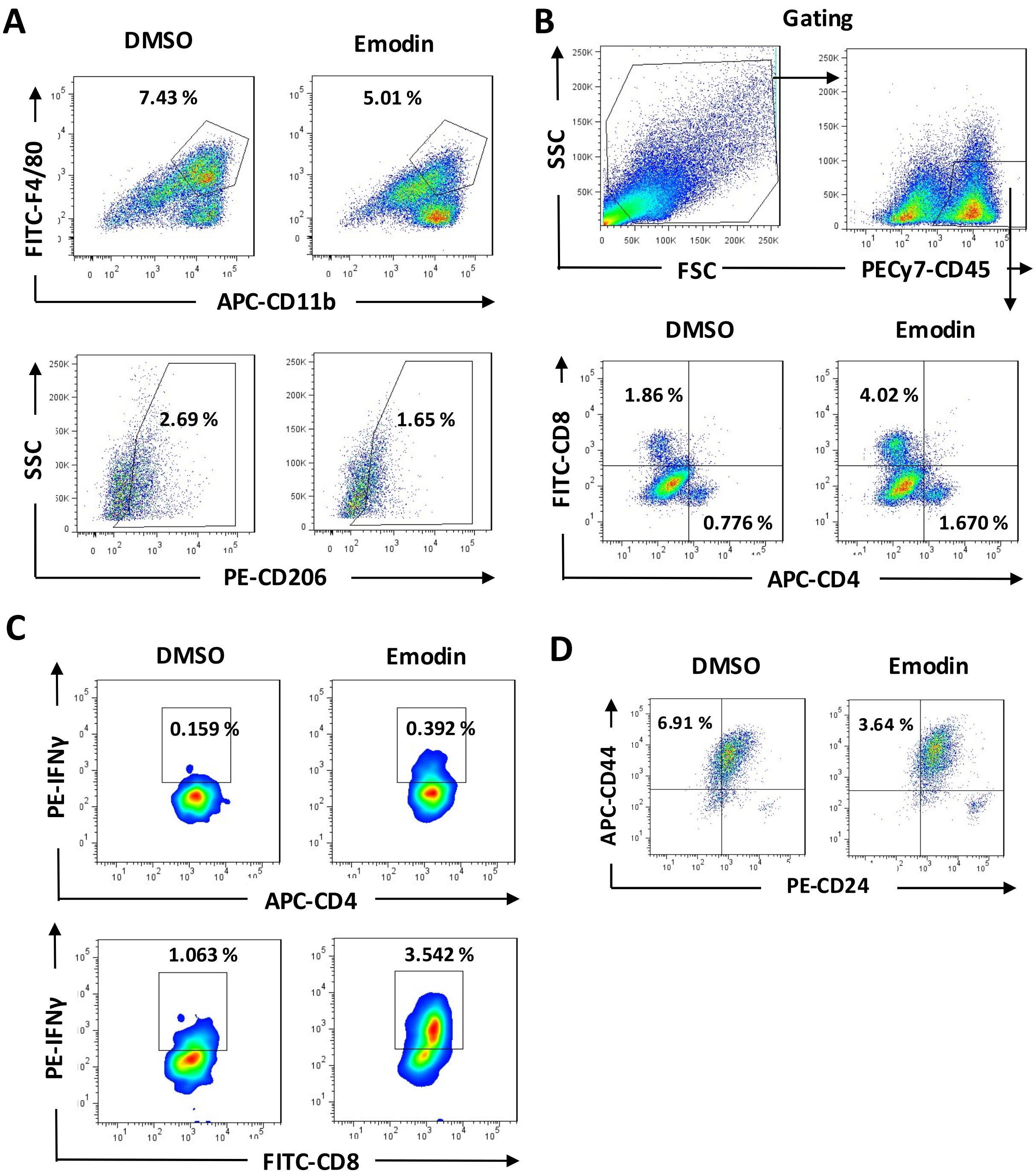

A
DMSO
Emodin
7.43 %
5.01 %
FITC-F4/80
APC-CD11b
2.69 %
1.65 %
SSC
PE-CD206
B
Gating
SSC
FSC
PECy7-CD45
DMSO
Emodin
1.86 %
0.776 %
4.02 %
1.670 %
FITC-CD8
APC-CD4
C
DMSO
Emodin
0.159 %
0.392 %
PE-IFNγ
APC-CD4
1.063 %
3.542 %
PE-IFNγ
FITC-CD8
D
DMSO
Emodin
6.91 %
3.64 %
APC-CD44
PE-CD24

Figure S8. Emodin enhances anti-tumor immunity and inhibits CSC in breast tumors

A. Representative flow cytometry images of total macrophage populations (CD11b+F4/80+) and CD206+ macrophages in 4T1 tumors. Cells were gated from the CD45+ cells in total cell suspension;

B-C. Representative images of total CD4+ or CD8+ T cells (**B**) and IFNγ+ producing T cells (**C**) in primary breast tumors;

D. Representative flow cytometry images of cancer stem cell (CD44+CD24-) in control and emodin treated tumors.

Figure S9.

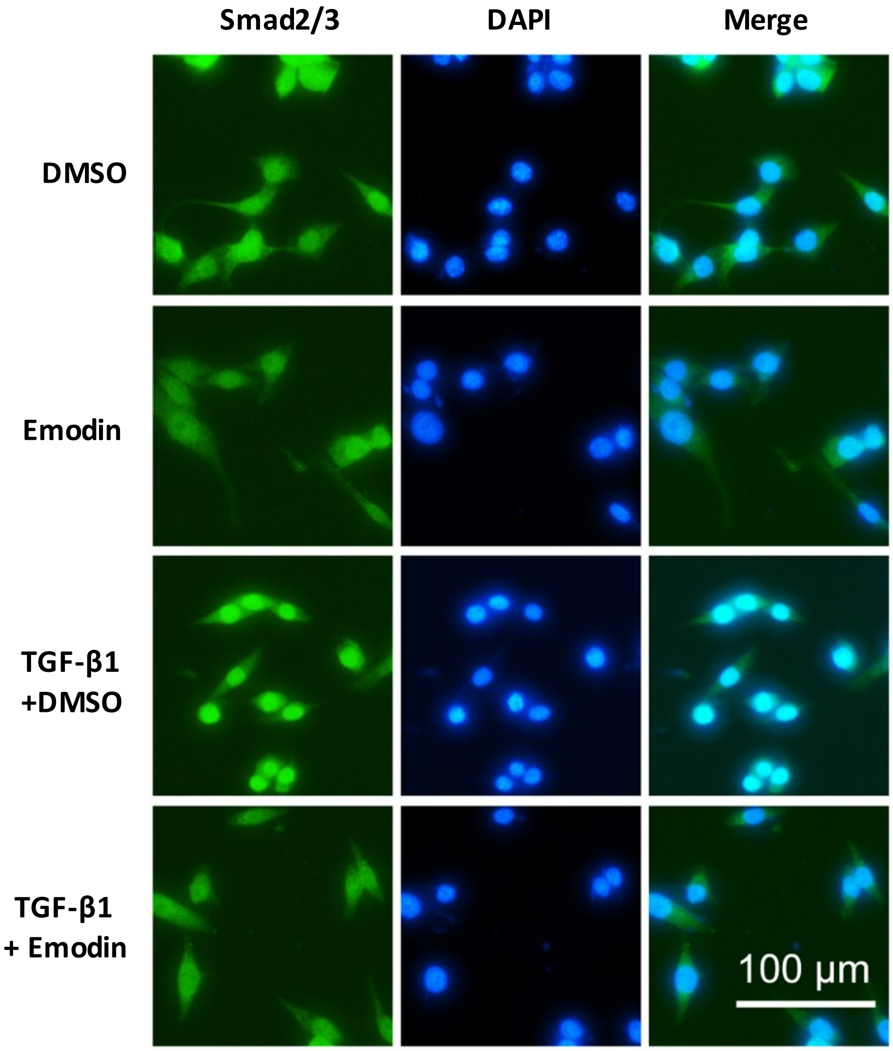

Figure S9. Emodin suppresses Smad2/3 nuclear translocation induced by TGFβ-1 in EO771 cells.

Representative images show the immunofluorescence staining for Smad2/3 in EO771 tumor cells following TGF-β1 stimulation with or without Emodin treatment (24 h).
